# Visual Receptive Field Properties of Neurons in the Mouse Lateral Geniculate Nucleus

**DOI:** 10.1371/journal.pone.0146017

**Published:** 2016-01-07

**Authors:** Jiaying Tang, Silvia C. Ardila Jimenez, Subhojit Chakraborty, Simon R. Schultz

**Affiliations:** Centre for Neurotechnology and Department of Bioengineering, Imperial College London, South Kensington, London, SW7 2AZ, United Kingdom; CSIC-Univ Miguel Hernandez, SPAIN

## Abstract

The lateral geniculate nucleus (LGN) is increasingly regarded as a “smart-gating” operator for processing visual information. Therefore, characterizing the response properties of LGN neurons will enable us to better understand how neurons encode and transfer visual signals. Efforts have been devoted to study its anatomical and functional features, and recent advances have highlighted the existence in rodents of complex features such as direction/orientation selectivity. However, unlike well-researched higher-order mammals such as primates, the full array of response characteristics vis-à-vis its morphological features have remained relatively unexplored in the mouse LGN. To address the issue, we recorded from mouse LGN neurons using multisite-electrode-arrays (MEAs) and analysed their discharge patterns in relation to their location under a series of visual stimulation paradigms. Several response properties paralleled results from earlier studies in the field and these include centre-surround organization, size of receptive field, spontaneous firing rate and linearity of spatial summation. However, our results also revealed “high-pass” and “low-pass” features in the temporal frequency tuning of some cells, and greater average contrast gain than reported by earlier studies. In addition, a small proportion of cells had direction/orientation selectivity. Both “high-pass” and “low-pass” cells, as well as direction and orientation selective cells, were found only in small numbers, supporting the notion that these properties emerge in the cortex. ON- and OFF-cells showed distinct contrast sensitivity and temporal frequency tuning properties, suggesting parallel projections from the retina. Incorporating a novel histological technique, we created a 3-D LGN volume model explicitly capturing the morphological features of mouse LGN and localising individual cells into anterior/middle/posterior LGN. Based on this categorization, we show that the ON/OFF, DS/OS and linear response properties are not regionally restricted. Our study confirms earlier findings of spatial pattern selectivity in the LGN, and builds on it to demonstrate that relatively elaborate features are computed early in the visual pathway.

## Introduction

The lateral geniculate nucleus (LGN) of the thalamus is strategically located within the visual system to modulate retinal afferents enroute to primary visual cortex (V1). Physiological properties of LGN relay cells play a key role in visual information processing along the visual pathway.

The LGN has traditionally been viewed as a passive relay station based on highly specified retinal ganglion cell (RGC) axons projecting to LGN, as well as similar receptive field properties of RGCs and LGN relay neurons. As proposed in the initial feed-forward model by Hubel and Wiesel [1], the only information accessible to V1 from subcortical thalamic neurons is the simple ON/OFF centre-surround receptive field characteristics, and all other properties are computed de novo in V1. For instance, the directional selectivity in V1 is a consequence of simple cells receiving inputs from several LGN neurons simultaneously leading to elongated ON/OFF subfields.

Nevertheless, the dual firing modes of thalamic neurons, burst and tonic [2,3], as well as various non-retinal inputs including cortical feedback and local interneurons, are among numerous pieces of evidence that have emerged in recent years suggesting that LGN is able to filter and introduce more complexity to retinal information before it reaches V1, and consequently operates as a “smart-gating” system for processing visual information. The LGN is therefore able to determine what, when and how information is delivered to V1 [4], and detailed physiological characterization of this thalamic nucleus can help in better understanding of higher visual function.

Compared to the well-characterized properties of monkey and cat LGN, mouse LGN has been largely neglected for visual research over the past decades. This is primarily due to its small brain volume and different organizational principles, such as lack of layer segregation, compared to higher-order mammals. However, highly developed genetic manipulations in mice provide powerful techniques for cell-type specific perturbation of firing patterns at single cell resolution [5,6]. Such endeavours may allow newer ways to address many open questions regarding neuronal processing in the visual network.

The past few years have witnessed an increased interest in the study of mouse LGN. The unique morphological features of mouse LGN relay neurons has been challenged by recent work revealing X- (biconical), Y- (symmetrical) and W- (hemispherical) cell subgroups according to their dendritic architecture and ocular specificity [7]. In addition to classical receptive field characterization studies of LGN neurons by Grubb and Thomson [8], Marshel and co-workers have shown the functional presence of relay cells in the superficial LGN that selectively respond to motion in the anterior-posterior axis [9]. This direction selectivity has been verified by two other studies subsequently [10,11]. Thus, there is ample evidence to suggest that LGN neurons, at least in mice, receive diverse inputs to process visual features before afferents reach V1.

## Materials and Methods

### Animals

Experiments were performed on adult (age 2–4 months) female C57Bl/6 mice (Harlan, UK). The animals were treated in accordance with the Animals (Scientific Procedures) Act 1986 (United Kingdom) and Home Office (United Kingdom) approved project and personal licenses, and the experiments were approved by the Imperial College Animal Welfare Ethical Review Board under Project License 70/7355. The mice were housed in the animal facility of Imperial College London under a 12-hour light/dark cycle. All electrophysiological recordings were carried out during the dark phase of the cycle.

### Anaesthesia

Each mouse was sedated with an intraperitoneal injection of chlorprothixene (Sigma-Aldrich, UK), at a dose of 0.5 mg/kg, to aid stress-free induction and reduce overall isoflurane concentration during the experimental procedure. After 10–15 min, the animal was transferred to the stereotaxic apparatus and positioned onto the incisor adaptor, facing the experimenter. Surgical anaesthesia was induced with 3–4% isoflurane in O_2_, and maintained with 1–1.5% isoflurane in O_2_ (Harvard Apparatus, UK) through a customized nose cone that provided minimal obstruction to visual stimulus presentation and stable recordings. Dexamethasone (2 mg/kg) (Organon, UK) and atropine (0.3 mg/kg, 20% in distilled water) (Animalcare, UK) were administered subcutaneously to improve breathing and reduce secretions respectively. Body temperature was monitored and maintained at 37±0.5°C by using a homeothermic heating device (Harvard Apparatus).

### Electrophysiology

The animal was moved to the recording setup and was placed on the stereotaxic apparatus facing straight towards the experimenter during surgery. Ear bars were securely positioned into the auditory canals. Following a scalp incision, a craniotomy of ~0.05 mm in diameter was performed over the cerebellum for securing the ground wire (silver). Typical recording coordinates were 2.15 mm lateral and 2.6 mm posterior from Bregma. For recordings aimed for the anterior and posterior LGN, the target coordinates were 2.15 mm lateral and 2.2 mm posterior from Bregma for anterior LGN, and 2.15 mm lateral and 3.0 mm posterior from Bregma for posterior LGN. The actual coordinates were scaled in cases where the Bregma-Lambda distance differed considerably from the average of 4.2 mm [12].

A circular rubber ring (~3 mm in diameter) was positioned around the desired recording site and glued with Histoacryl (B. Braun, Germany). A headplate was screwed on the stereotaxic apparatus, and dental cement was subsequently used to cover the skull and secure the headplate, leaving out the space inside the rubber ring. Once the dental cement cured, the ear bars were removed and the mouse was kept in place with the headplate. The stereotaxic platform was then rotated, with the mouse facing the monitor for recording purposes.

A craniotomy was performed over the area within the rubber ring. An MEA (A1x16-Poly2-5mm-50s-177-A16, NeuroNexus) was vertically inserted into the brain through the craniotomy using a microdrive (Patchstar, Scientifica, UK). Before reaching the LGN, at a depth 2,000–2,200μm below the pia, the electrode revealed 400–500μm wide high-frequency hippocampal activity. LGN was usually found at a depth of 2,500–3,200 μm from the pial surface, characterized by robust visual responses, either to gratings or to white/black squares. Once the electrode was confirmed to be in LGN, with most channels obtaining reliable visual responses, it was allowed to settle in position for ~30 min to obtain stable recordings. Signals were amplified (gain = 6000) (Lynx-8, Neuralynx, USA), and recorded using a CED 1401 and Spike 2 software (version7 Cambridge Electronic Design, UK) on a PC. The signal was sampled at 20 kHz and band-pass filtered between 300 Hz and 9000 Hz, to extract spiking activity.

Skull and brain tissue were kept moist with sterile phosphate buffered saline (PBS) throughout the experiment. A thin layer of silicon eye oil (viscosity 100 cSt, Sigma-Aldrich, UK) was applied on the animal’s eyes. This was applied often during the experiment to prevent corneal dehydration while maintaining a clear optical transmission medium. All recordings were performed in a dark room. The monitor presenting the visual stimuli was the only source of illumination to the animal.

At the end of the recording, the electrode position in X-, Y-, and Z-axis was recorded from the microdrive, and the electrode was withdrawn and soaked into DiI (Sigma Aldrich, UK) /DMSO (Sigma Aldrich, UK) solution (20 mg DiI in 300 μl DMSO) for a few seconds, and left to dry. The electrode was then inserted quickly to the same recording coordinates in the brain, taking about 5–10 seconds to reach the desired depth for recording, and then withdrawn slowly after having settled in the brain for approximately 25 min. The time allowed DiI to fully spread among neurons around the electrode shank. Following an overdose of 10% urethane (Sigma Aldrich, UK), the animal was transcardially perfused with PBS (Life Technologies, UK) and fixed with 4% paraformaldehyde (PFA) (Sigma-Aldrich, UK) solution. The brain was dissected out and post-fixed overnight in 4% PFA at 4°C, and then transferred to PBS solution at 4°C until further histological processing.

### Stimulus presentation

Stimuli were displayed on a Samsung SyncMaster 2233RZ monitor (22” LCD monitor, 120 Hz refresh rate), which has been reported to possess temporal reliability for visual research [13]. The monitor was gamma corrected and stimuli were displayed using a 255 grey level scale. The mean screen luminance was 46.93 cd/m^2^.

The monitor was placed 25 cm from the animal, providing a viewing angle of approximate ~50 deg on each side from the centre of the monitor. Hand mapping of receptive field was firstly carried out in 2–3 nonadjacent channels in most of the experiments, using Expo visual stimulus software developed by P. Lennie (https://sites.google.com/a/nyu.edu/expo/). Real-time neuronal responses were monitored using a customized 16-channel audio splitter, with the main component as a 16-channel-Multiplexer.

A battery of stimuli was used to characterize a wide variety of response features of the mouse LGN cells. For electrophysiological characterization, all stimuli were presented using the software Flymouse. Flymouse was customized from a MATLAB Psychophysics Toolbox (MathWorks) based interface developed by the Motion Vision Group at Uppsala University (http://www.flyfly.se/about.html). Sinusoidal monochromatic drifting gratings, covering the full extent of the monitor, were used for recordings. The parameters used for each grating stimulus are listed in Table 1. Apart from sinusoidal gratings, flicker and a contrast noise movie were also presented to the animal.

**Table 1:** Parameters of sinusoidal grating stimuli for each stimulus set.

Parameter	Spatial Frequency Set	Temporal Frequency Set	Contrast Set	Direction Set
Spatial frequency (c/deg)	0.02; 0.03; 0.04;	0.03	0.03	0.03
	0.06; 0.08; 0.12;			
	0.16; 0.32; 0.64;			
	0.96			
Temporal frequency (Hz)	1	0.3; 0.4; 0.6;	1	1
		1.2; 1.6; 2.4;		
		3.2; 4.8; 6.4;		
		9.6		
Contrast	0.98	0.98	0.10; 0.20; 0.39;	0.98
			0.58; 0.78; 0.98	
Direction (deg)	0	0	0	0; 45;
				90; 135;
				180; 225; 270;
				315
Number of trials	60	40	60	48
Individual trial duration (sec)	7	14	7	7
Trial repetition	6	4	10	6
Pseudorandom sequence	Yes	Yes	Yes	Yes
1 sec blank screen before and after each trial	Yes	Yes	Yes	Yes

The flicker stimulus consisted of a full field screen, black (minimum luminance) or white (maximum luminance), presented for 600 msec. Black and white trials were interleaved with a total of 800 repetitions per session. This stimulus was used to assess transient or sustained responses.

A contrast-modulated noise movie [14] was used to systematically map receptive fields. This stimulus was presented using Flymouse. In brief, the contrast noise movie was a spatiotemporal noise movie multiplied by a 0.1 Hz sinusoidally varying contrast modulation to prevent response adaptation. The noise movie was created in the Fourier domain to limit spatial and temporal properties of a random three-dimensional (3D) spectrum and was then converted to the temporal domain. The spatial frequency spectrum drop off was set at A(f) ~ 1/(f+fc) (fc = 0.05 c/deg) and with a spatial cutoff at 0.16 c/deg, and a temporal frequency spectrum flattened with a sharp low-pass cutoff at 10 Hz. Each session began with a 5 min presentation of grey background and followed by a 5 min of the movie. The whole session was presented four times, total duration being 40 min, enough to trigger a sufficient number of spikes to ascertain the receptive field using a spike-triggered average [10,14].

### Histology

On the day of imaging, an incision was first made on the contralateral brain with a blade to help with site localization. The brain was then embedded with 2% agarose (Sigma Aldrich, UK), and sectioned coronally at 200 μm on a vibrating Microtome (VT1000 S, Leica Microsystems), at a speed 0.65 mm/sec and a frequency of 50 Hz.

The sections were then mounted on positively charged slides (Thermo Scientific, UK). Following incubation with DAPI (Fluoroshield with DAPI, Sigma Aldrich) for 15–20 min to stain nuclei, sections were covered with coverslips (Thermo Scientific, UK). Afterwards, slides were kept away from the light for a few hours for DAPI to dry. Sections were imaged on Leica TCS SP5 with 5x and 10x air objectives.

The outlines of the LGN and the electrode track from confocal microscopy were traced manually. The boundaries of the LGN were readily identifiable from DAPI staining [10,15].

### 3D histological reconstruction of the LGN

To precisely map the position of electrode track and recorded neurons in LGN volume, a 3D LGN model was constructed using a histological approach, to which the confocal image was mapped to characterize neurons as locating within the anterior, middle or posterior LGN. The protocol is detailed below.

Sudan Blue II (Sigma-Aldrich, UK) was selected for staining individual brains due to its propensity to mask fluorescent structures [16]. Sudan Blue II was mixed with paraffin wax (Fisher Scientifica Ltd) and kept in the oven (Genlab, UK) at 60°C till the wax fully melted to produce a 2% Sudan Blue II solution. Thorough stirring was crucial here to avoid accumulation of crystal that could degrade the quality of the sections and subsequent imaging. The brain sample went through dehydration, staining and embedding procedures, according to the following protocol.

Day 1:

1. Dehydration

70% ethanol 30 min

90% ethanol 30 min

100% ethanol 45 min

100% ethanol 45 min

100% ethanol 30 min

2. Cleaning

Histoclear 30min

Histoclear 30min

3. Cleaning + Dye infiltration

Histoclear + 3% Sudan Blue II (0.3 g Sudan Blue II in 10 ml histoclear) for 2–3 hours at room temperature.

4. Dye infiltration

2% Sudan Blue II in paraffin wax inside embedding oven (TAAB MK II) at 60°C with a pressure of 600 mBar overnight.

Day 2—Embedding:

Using a pre-warmed KD-BM Tissue Embedding Centre (Jinhua Kedi Co. Ltd.), the brain specimen was embedded in 2% Sudan Blue II in paraffin wax, in an embedding ring (Fisher Scientific Ltd, UK), and was left to cool down and mould.

After waiting for at least 4 hours for the specimen to fully settle in the wax, the specimen block was trimmed with a microtome (Leica 2655) at speed 3 and thickness of 10μm/slice until the specimen position was recognisable and a smooth wax surface obtainable. The wax around the specimen was manually trimmed with a blade, leaving the specimen area sticking out, and finally, the specimen module was ready to be processed with the histocutter.

The Histocutter [17] is a purpose-built robotic device for high-throughput 3D tissue visualization, whereby, multi-spectral signals can be imaged from thousands of aligned tissue slices, with thickness as thin as 1 μm. The histocutter and its corresponding Java-based CutterMaster software used in this project (originally developed by J. Crawford and J. Reynaud at Devers Eye Institute) was a duplicated system in use at the Osteoarthritis Centre of Excellence, Imperial College London, and now under development and maintenance in the Department of Bioengineering, Imperial College London.

On the Histocutter, the specimen was trimmed for a few sections with the microtome for blade alignment. Focus position was decided under the facilitation of the objective lens on the microscope and real-time updating of images from the Streaming function on the CutterMaster. Subsequently, the microtome was switched to the continuous section mode, with the number of slices set at 300 on the CutterMaster. The system was left for processing overnight (Settings: Cutting speed: 3; Knife angle: 2.5; Lamp intensity: 100; Channel No.: 2 (Excitation at 560 nm, Emission at 645 nm); Thickness: 5 μm/slice; Focus exposure (sec): 5; Thumbnail Exposure (sec): 20; Objective lens (from Nikon) and zoom: AZ Plan Fluor 2x, Zoom 2x, resulting in a working distance of 45 mm., Field of view (mm): 4.585, Microns per pixel: 1.119).

The image stack was opened in the Amira software (version 5.2.2) after being downscaled to 512×512 pixels on ImageJ, with voxel size: x: 4.476; y: 4.476; z: 5. The LGN volume was reconstructed by manually segmenting the LGN boundary every 5–10 voxels through the entire LGN. Segmentation and eye-inspection were repeated on voxels that either failed to match with the original image or had inconsistent shapes, until a 3D LGN volume with a satisfying smooth edge was obtained.

The LGN volume representation was evenly divided into three sub-divisions along the anterior-posterior axis, named as anterior, middle and posterior LGN respectively. For the image taken with confocal microscopy after each recording, it was adjusted to the same resolution as the 3D LGN on ImageJ, and then imported to the Amira at voxel size: x: 4.476; y: 4.476; z: 1. A continuous comparison between the confocal image and the 3D LGN along the anterior-posterior axis was performed mainly on the morphological features, until the best match was found throughout the LGN volume. Based on its relative location within the 3D LGN, electrode and single cell locations were categorized as being in the anterior/middle/posterior LGN.

### Data pre-processing

Single-units and multi-units were identified and isolated by custom routines in MATLAB and Klusta-Kwik [18]. Quality of single unit separation was checked based on a clear refractory period, cross-correlogram, and discriminative features like waveform shape and amplitude when compared to neighbouring clusters.

In addition, stability of single units was assessed to exclude occasions where electrode shift or other mechanical damage was suspected. Units that suffered from significant amplitude and/or waveform changes, or stopped responding during presentation of stimuli were not included in the analysis. Subsequent analyses were carried out with custom-written MATLAB routines.

Histology and confocal imaging were performed after recording (procedures described in the previous sections). Units from electrode sites found outside the edge of the LGN were excluded from subsequent analysis.

### Data analysis

Peri-stimulus time histograms (PSTH) were used to characterize responses to different stimuli. Responses to gratings were fitted to classical response functions (see below) using least-square error minimization. Receptive fields were obtained by inverse correlation or spike-triggered averaging (STA), and then fitted to a 2-dimensional (2D) Gaussian function,

$$\mathrm{RF}\left(x,y\right)={\mathrm{Ae}}^{-\left(\left(\frac{x-x_{c}}{2\sigma_{x}^{2}}\right)-\left(\frac{y-y_{c}}{2\sigma_{y}^{2}}\right)\right)}$$ Eq. 1

where A is the intensity of the response, (*x*_*c*_, *y*_*c*_) are the coordinates for the peak, and *σ*_*x*_ and *σ*_*y*_ correspond to the standard deviation of the Gaussian function in the *x* and *y* directions, and are taken as the estimates of the radii of the function in each direction.

Spatial frequency properties were assessed by presenting vertical drifting gratings of various spatial frequencies ranging from 0.02 to 0.96 c/deg Gratings were presented at 1 Hz temporal frequency and 0.98 contrast level. Spatial frequency response (spatial frequency versus firing rate) was fitted to a difference of Gaussian (DoG) equation function [19,20].

$$R(v)=\;b+(k_{c}-b)(e^{-{\left(\pi r_{c}v\right)}^{2}}-k_{s}e^{-{\left(\pi r_{s}v\right)}^{2}})$$ Eq. 2

where *R* is the firing rate, *v* is spatial frequency, *b* is the baseline firing rate, *k*_*c*_ is the area under the receptive field’s centre Gaussian, *k*_*s*_ is the relative area under the receptive field’s surround Gaussian function, *r*_*c*_ and *r*_*s*_ are the radii of the centre and surround Gaussian functions respectively, at the point where each mechanism reaches 1/e of its peak.

The preferred spatial frequency was taken as the frequency where the fitted function reached its peak amplitude, and the spatial frequency cutoff is taken as the spatial frequency at which the response amplitude reached 1% of its peak amplitude [8].

Temporal frequency tuning was calculated with sinusoidal gratings of a series of temporal frequencies from 0.3 to 9.6 Hz, at 0.03 c/deg spatial frequency with 0.98 contrast level. The tuning curve was fitted with a two-Gaussian-halves function [8].

$$R\left(\omega \right)=\;b_{1}+\left(a-b_{1}\right)e^{-{\left[\frac{p-\omega }{s}\right]}^{2}}\;\mathrm{for}\;\omega <p$$ Eq. 3

$$R\left(\omega \right)=\;b_{2}+\left(a-b_{2}\right)e^{-{\left[\frac{p-\omega }{s}\right]}^{2}}\;\mathrm{for}\;\omega >p$$ Eq. 4

where *R* is the firing rate, *b*_1_ and *b*_2_ are the baselines of the low- and high-frequency sides of the function respectively, *a* is the response amplitude at the optimal temporal frequency, *ω* is the temporal frequency, *p* is the peak temporal frequency, and *s* is the standard deviation of the Gaussian.

Each unit’s preferred temporal frequency, low_50_ and high_50_ (low_50_ and high_50_ represent the temporal frequencies at which the response amplitude reached 50% of its maximum before and after the peak response respectively), and tuning bandwidth (difference between high_50_ and low_50_) were measured from the fitted curves described above.

Contrast response was assessed with a drifting grating at 0.03 c/deg spatial frequency and 1 Hz temporal frequency with varying contrast levels. Plots of stimulus contrast versus firing rate were fitted with a hyperbolic function [21].

$$R\left(c\right)=\;b+\left(R_{\max}-b\right)\frac{c^{n}}{c^{n}+h^{n}}$$ Eq. 5

where R is the firing rate, *c* is the contrast, *R*_*max*_ is the maximum response, *h* is the contrast at which the response reached 50% of its maximum, and *n* is the rate of change of firing rate with respect to contrast.

We measured the contrast gain as the slope of a tangent to the curve where the amplitude of the cell’s response was 20% of its value at full contrast. In addition, we calculated C_50_ that was viewed as the contrast at which the response amplitude fell to 50% of its value at full contrast.

Direction and orientation selectivity were estimated from the responses to a drifting sinusoidal grating moving in eight evenly spaced directions, at 0.03 c/deg spatial frequency, 1 Hz temporal frequency and with a 0.98 contrast level. Spontaneous activity was first subtracted from the overall responses. The orientation and direction selectivity indices were obtained as the absolute value of the two equations given below. Preferred orientation and direction was calculated as the phase of the same equation.

$$\frac{\sum \;F\left(\theta \right)e^{2i\theta }}{\sum \;F\left(\theta \right)}\;\mathrm{for OSI and}\;\frac{\sum \;F\left(\theta \right)e^{i\theta }}{\sum \;F\left(\theta \right)}\;\mathrm{for DSI}$$ Eq. 6

Units with DSI > 0.33 were classified as direction selective, and units with OSI > 0.6 were defined as orientation selective.

Linearity was computed from the same stimulus used to probe spatial frequency properties. Spontaneous activity was first subtracted. The discrete Fourier transform was then applied and F1/F0 was computed from responses to gratings moving at the spatial frequency closest to each unit’s preferred spatial frequency. F1/F0 is the ratio of the first harmonic (response at the drift frequency) to the zeroth harmonic (mean response) [22]. A high F1/F0 ratio indicates that the cell responds to sinusoidal grating with a sinusoidal output at the stimulus temporal frequency. A low F1/F0 ratio represents a relatively continuous firing throughout the presentation of the grating. We define F1/F0 = 1 as the threshold for linearity.

Responses to full-screen black and white flicker were used to assess the sustained or transient response of an individual unit. The transient/sustained index is calculated as the ratio of the average firing rate during the first 50 msec. of the response to the average firing rate of the remaining response duration. Units were classified as sustained if the index value fell below 1, indicating that responses were maintained over the entire duration of stimulation. Transient cells were units with transient/sustained index above 1, whose initial response to stimulus onset decreased after 50 msec.

### Statistics

Normality was assessed with a D’Agostino-Pearson test in data sets that were large enough (n>30). Normally distributed data were described by mean ± standard error of the mean (SEM) and compared with unpaired *t*-tests. Non-normally distributed data were presented in the format of medians (25 percentile, 75 percentile), and the Mann-Whitney-Wilcoxon test was used for comparison between two groups, while for non-parametric comparisons of three or more groups a Kruskal-Wallis test was used. All comparison tests were two-tailed, and the level of significance was set at 0.05 unless specified. In figures, **** signifies *P*<0.0001, *** *P*<0.001, ** *P*<0.01, *P<0.05.

## Results

Visual response properties of mouse LGN were investigated using a multi-electrode array targeting the LGN, and neuronal discharge patterns were assessed with a variety of visual stimuli. Using such a method, we were able to record simultaneously multiple single unit responses and compare their properties in a single subject. The anatomical locations of putative neurons were then determined in 3D coordinates using a new reconstruction technique detailed in the Materials and Methods section.

For each animal, the number of penetrations was limited to three to maintain viability of brain tissue. From a total of 20 mice, 28 penetrations successfully targeted the LGN. Following histological confirmation of electrodes residing within the LGN, we performed unsupervised and manual clustering of the data to ascertain single cell responses. We found 189 well-isolated neuronal units that displayed robust centre-surround receptive fields. Receptive fields were reconstructed using STA analysis (see Materials and Methods). The number of units in each recording varied from 3–12. A small subset of units were excluded from our dataset due to their lack of response to any episodic drifting grating stimuli (4/189, 2.1%). After these exclusions, 185 units were used to characterize response features of the LGN.

To explore a broad spectrum of visual responses, we used a set of stimuli that allowed the study of classical and non-classical response features. We began with investigating the receptive field structure of LGN units in the following ways.

Receptive field profiles were extracted from responses to the contrast noise movie stimulus using the STA technique. All cells included in the data set displayed classical centre-surround receptive field structures similar to previous LGN studies [8,23].

**Fig 1:**
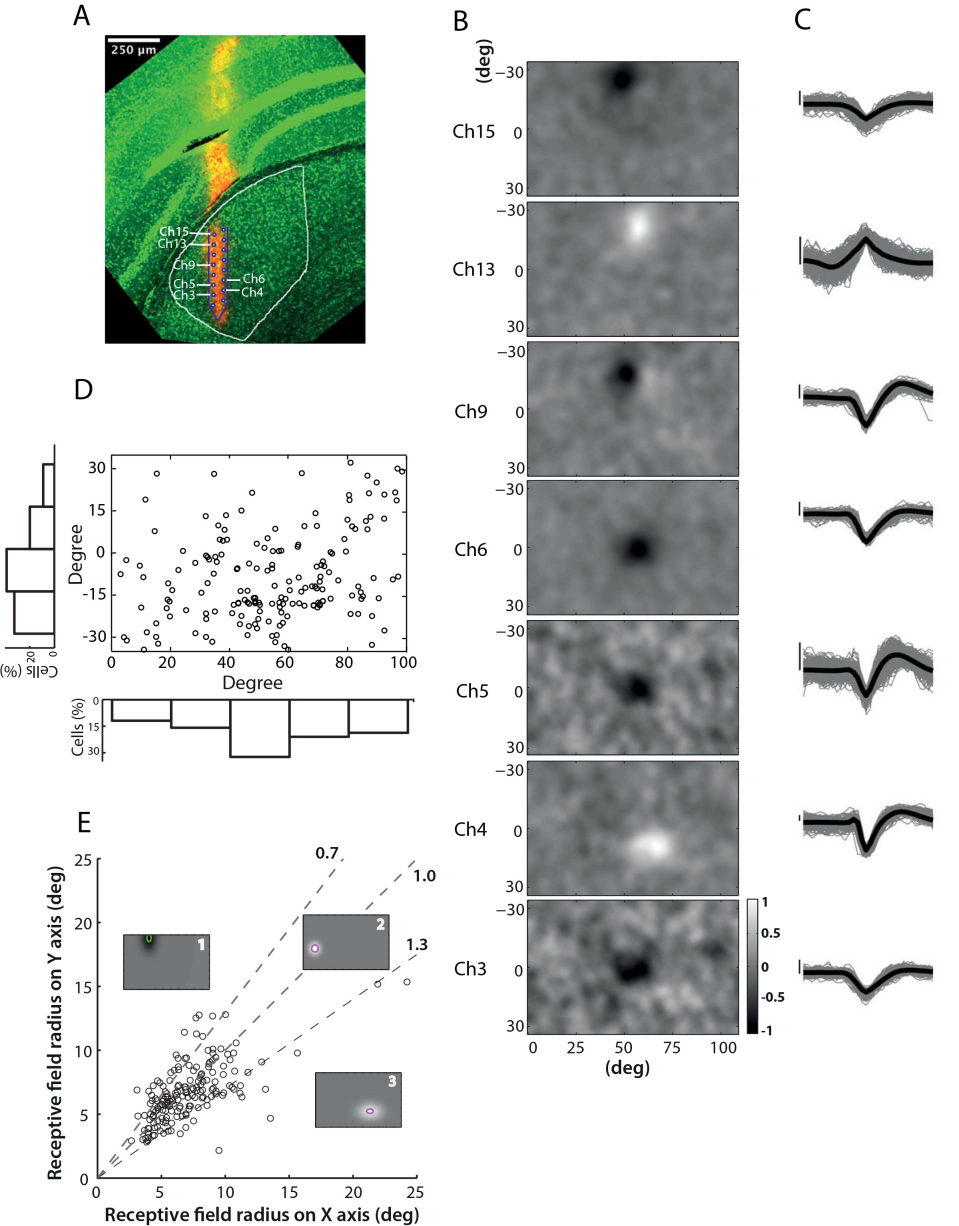
Spatial receptive field profiles of mouse LGN neurons. A) Confocal image of a coronal section of a brain slice (200 μm thickness). The electrode was dyed with DiI/DMSO solution (red track), and the slice stained with DAPI (Diamidino-2-phenylindole, displayed in green). The LGN boundary and electrode track within the LGN are delineated in white and purple respectively. Electrode sites corresponding to seven recorded neurons are labelled. B) 2D profiles of receptive fields from seven units reconstructed with the STA technique. The colour map in grey-scale shown at the bottom right indicates the intensity of the response of each unit to the contrast-noise movie (see Methods). MEA channel labels correspond to those in the histological image (A). C) Spike waveforms for the neurons shown in (B). Vertical scale bars indicate 25 mV. D) Receptive field centre location for all units in the dataset. The main panel displays the receptive field location of each cell within the visual display, with histograms at left and bottom showing the distributions of receptive field locations on the vertical and horizontal axis respectively. E) Correlation between receptive field radius vertical and horizontal components. Three single-cell representatives show cells whose receptive field fell into the three categories respectively: larger size on the vertical axis (1), circular (2) and larger size on the horizontal axis (3).

Neuronal units were classified as ON (n = 90; 48.6%) or OFF (n = 95; 51.4%) subclasses based on their receptive field polarities. Fig 1B shows spatial receptive fields from one such recording; the corresponding waveforms for each unit are shown in Fig 1C. The location and size of each receptive field are shown in comparison to the grey background representing the whole field of view that was covered by the stimulus. Histological visualization of the electrode track confirmed the spatial localization of the units within the LGN. It is observable from the reconstruction (Fig 1A), that units from this recording display spatial alignment with the electrode configuration. The receptive fields are located towards the middle of the stimulated section of the visual field, and are aligned from the top to the bottom in the same direction as the electrode in the LGN.

Fig 1D presents the location of the receptive field centres for all units within the field of view covered by the monitor, with population distributions shown by histograms at the bottom and left. The majority of the cells reveal receptive field centres located within -30~0 deg (Y axis) and 40~100 deg (X axis) in the visual dimensions of the monitor. Such a distribution indicates that the population of LGN cells recorded spans most of the stimulated visual field in our data set. Interestingly, numerous cells have receptive fields around the bottom centre of the monitor due to over-sampling of the representative area of the LGN. These cells, however, show a retinotopic organization, and probably is an outcome of placement of electrodes targeting the middle LGN.

To evaluate how much the receptive fields differ from classical circular receptive fields, we calculated the radii along both horizontal and vertical axes for each unit. The radii were estimated by fitting the data to a 2D-Gaussian function and measured as the standard deviation of the Gaussian in the *x*- and *y*-dimension. We then calculate the ratio between the horizontal radius and the vertical radius in order to assess whether the receptive fields are circular. As indicated in Fig 1E, cells with the radius ratio between 0.7–1.3 were defined as circular cells; while those outside this range were categorized as non-circular, (two dash lines, with the middle dash line indicating 1). Based on this criterion, 75.1% (139/185) of the units in the data set displayed circular receptive fields. For these units the absolute radius was measured as the average between the radius on the horizontal- and vertical-axes. Within this sub-population, there were 3.6% (5/139) units with receptive field radius exceeding 10 deg, with a maximum radius of 11.5 deg, and the median with interquartile ranges of receptive field centre radius was 6.5 (5.3, 8.1) deg.

### Spontaneous and evoked activities

We examined the spontaneous and evoked firing rates of mouse LGN neurons. These properties serve as the first step to study the activities of cells in the LGN and explore the extent of their responsiveness to visual stimuli, and provide a link for comparison with previous studies.

Spontaneous activity was assessed from responses to a grey full field stimulus at mean luminance (46.93 cd/m^2^), whereas evoked activity was measured as the maximal response to any grating stimulus.

**Fig 2:**
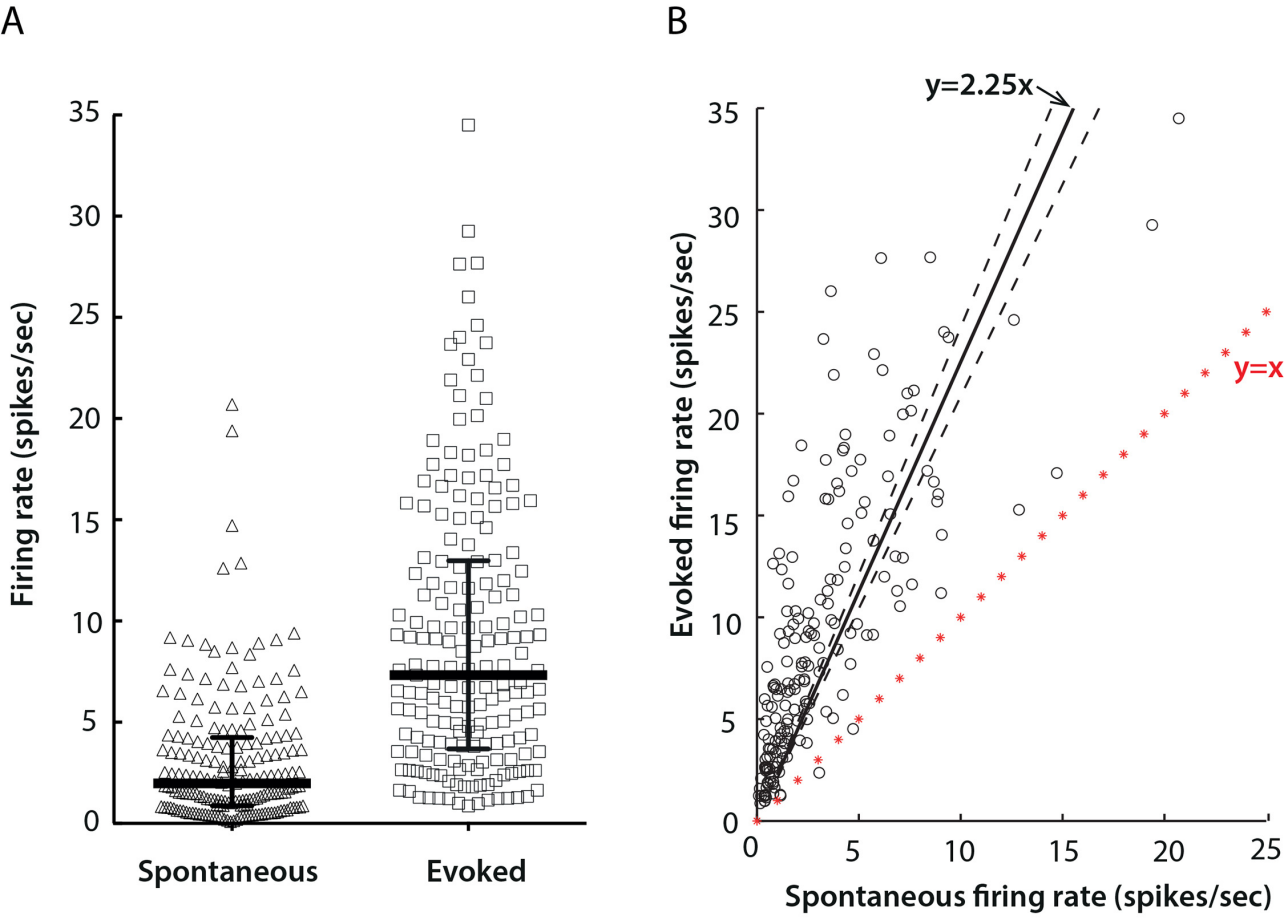
Spontaneous and evoked firing rates of mouse LGN neurons. A) Distributions of spontaneous (triangle) and evoked (square) firing rates of single cells. Solid lines show medians with interquartile ranges; the medians were 2.0 spikes/sec and 7.3 spikes/sec for spontaneous and evoked firing rates respectively. The horizontal position of data points is for clarity in showing the distribution, and conveys no further meaning. B) Correlation of spontaneous (x) and evoked (y) responses. The red line marked with stars represent y = x. Solid line shows the least squares best-fit line, y = 2.25x, and dashed lines the 95% confidence interval 2.08–2.42. *R*^2^ = 0.31.

Most of the units (149/185, 80.5%) fired at a frequency below 5 spikes/sec during the spontaneous condition, while the evoked response covered a broader range and reached as high as 35 spikes/sec. The evoked firing rate of the majority of units (183/185, 98.9%) was above the corresponding spontaneous firing rate. Across the population, the median with interquartile ranges of spontaneous firing activity was 2.0 (0.9, 4.3) spikes/sec, while the median with interquartile ranges of the evoked response was 7.3 (3.7, 13.0) spikes/sec (Fig 2A). Statistically, the evoked activity was approximately 125% higher than the baseline response (y = 2.25x, with a 95% confidence interval of 2.08–2.42, and *R*^2^ = 0.31) (Fig 2B).

### Spatial frequency tuning properties

Spatial frequency tuning properties were obtained from responses to drifting gratings of various spatial frequencies, and fitted with a Difference of Gaussians (DoG) function [19,20]. A total of 92 out of 185 units in the data set were well fitted (i.e. the fit explained at least 95% of the variance in the response) and were included in subsequent spatial frequency tuning analysis.

**Fig 3:**
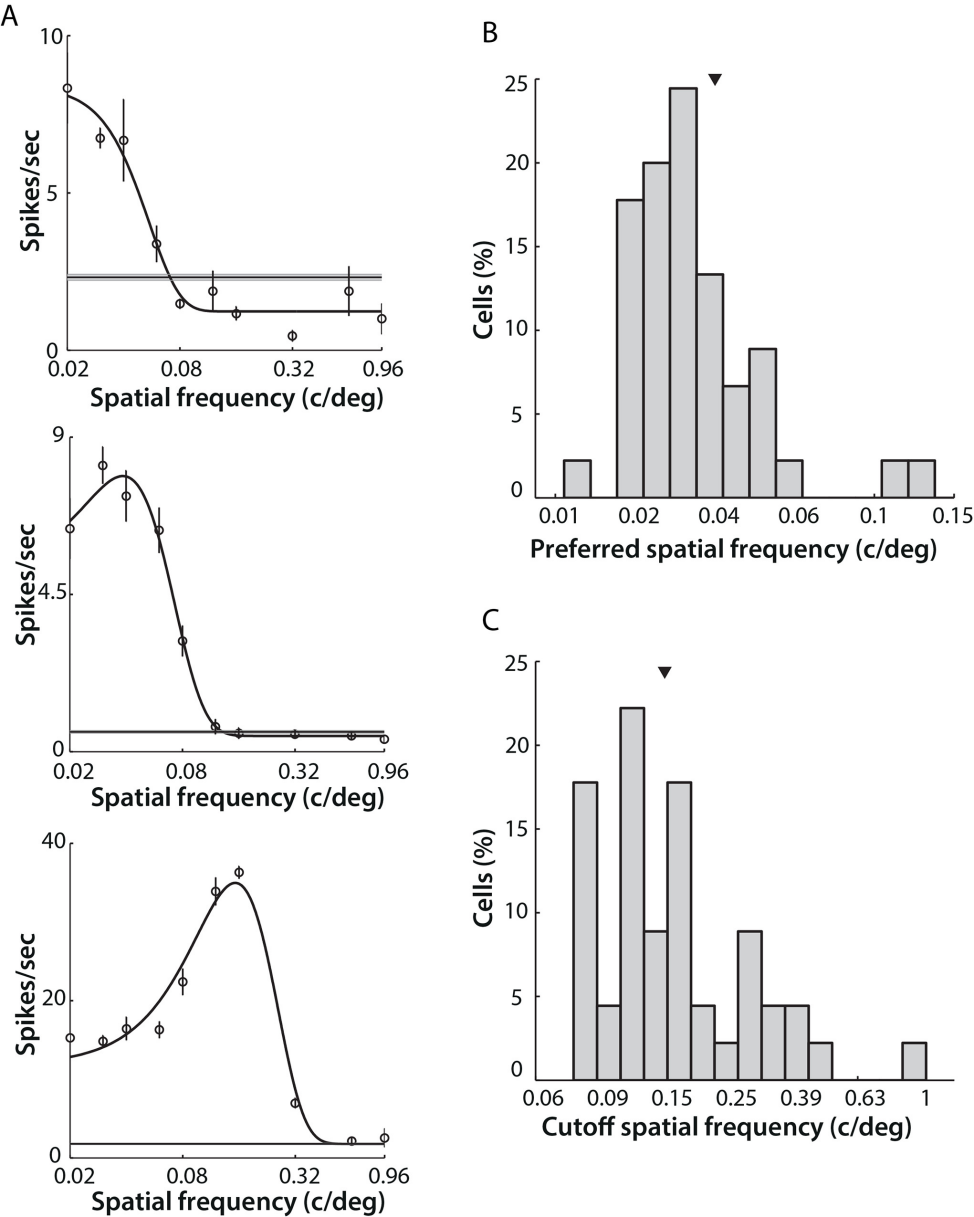
Spatial frequency tuning properties of mouse LGN cells. A) Examples of spatial frequency tuning of three single cells. Open circles indicate mean firing rates and error bars indicate SEM across six repeated presentations of the drifting gratings. Black curves show the best fits of these raw data to a DoG function. The grey area indicates the SEM of spontaneous activity, with thinner lines indicating mean values. Note: logarithmic scale of x-axis. Top left, a typical cell showing a “low-pass” tuning response. Middle panel: a “band-pass” cell. Bottom left, a “bandpass” cell preferring higher spatial frequencies. B) Distribution of preferred spatial frequency for bandpass cells (45 cells of 92). Median (arrow) = 0.035 c/deg. C) Distribution of cut-off spatial frequency for bandpass cells. Median (arrow) = 0.16 c/deg.

Units in the LGN generally displayed all three types of spatial frequency selectivity: low-pass, high-pass, and band-pass. The example shown at top in Fig 3A, defined as “low pass” filtering, represents the most common response type: cell activity progressively decreased as the spatial frequency increased, starting from the lowest spatial frequency value. However, we could not distinguish whether cells in this category were truly responding as low pass filters, or if instead they actually preferred extremely low spatial frequencies (lower than 0.02 c/deg). The example shown in the middle of Fig 3A reveals a “band pass” response type: cell activity was maximal at ~0.04 c/deg and decreased rapidly as spatial frequency further increased. The bottom example in Fig 3A illustrates a cell that responded best to a higher spatial frequency, over 0.10 c/deg. The preferred spatial frequency of this cell was 0.13 c/deg with cut-off frequency (taken as the high spatial frequency at which response amplitude reached 1% of its maximum) at 0.45 c/deg.

LGN cells typically preferred very low spatial frequencies, with over half of the neurons in the dataset (47/92, 51.1%) showing “low-pass” characteristics. These units were excluded from the calculation of preferred and cut-off spatial frequency. The median preferred spatial frequency was 0.035 (0.0307, 0.045) c/deg (Fig 3B). The cut-off frequency, which represents the highest spatial frequency that LGN cells are capable of resolving, was relatively low in mice, with a median of 0.16 (0.13, 0.25) c/deg (Fig 3C).

### Temporal frequency tuning properties

Temporal frequency response properties were ascertained by fitting data to a 2-Gaussian-halves function [8]. 58 units were included in the analysis using the same criterion as with spatial frequency analysis.

**Fig 4:**
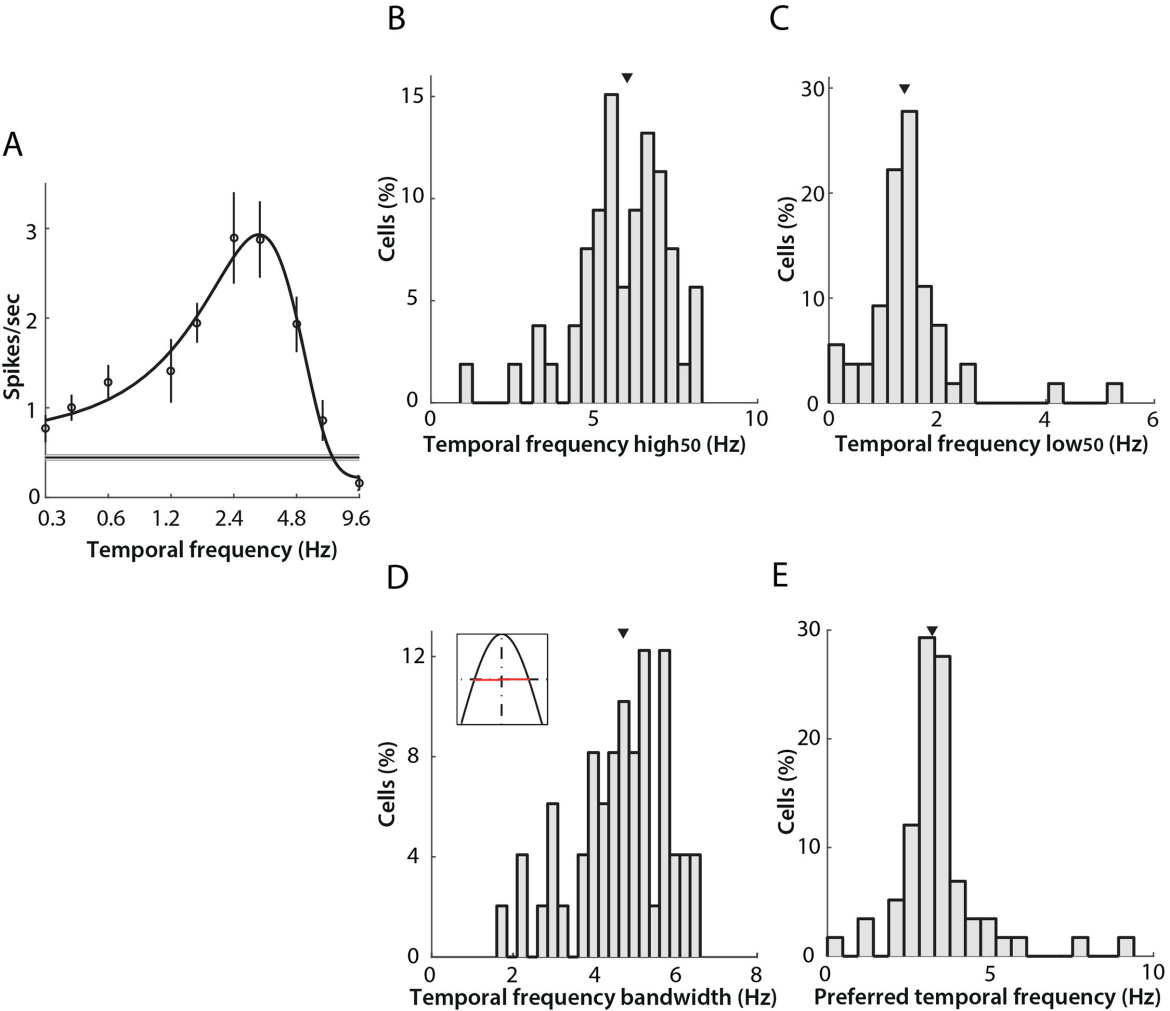
Band-pass temporal frequency tuning properties of neurons in mouse LGN. A) An example of commonly encountered temporal frequency tuning profile (“band-pass”). Open circles indicate mean firing rates, and error bars the SEM across four drifting grating repeats (see Table 1). The black curve shows the best fit of a two-half-Gaussian function. The grey area indicates the SEM of spontaneous activity, with the thinner dark line indicating the mean value. Panels B-E present additional parameters measured from the band-pass subtype that represent 84.5% (49/58) of neurons in the data set. In all cases, the arrows show the median of the distribution. B) Distribution of high_50_ cut-off, with median 6.0 (5.3, 7.0) Hz. C) Distribution of low_50_ cut-off, with median 1.40 (1.07, 1.60) Hz. D) Distribution of tuning bandwidth, calculated as the difference between high_50_ and low_50_ (range illustrated in red in the inset) with median 4.70 (4.07, 5.60) Hz. E) Distribution of preferred temporal frequency with median 3.2 (2.9, 3.6) Hz.

The temporal frequency tuning of individual LGN units was categorized into three subtypes as before, although bandpass characteristics were found most commonly. A typical example of band-pass tuning is shown in Fig 4A; firing rate increased steadily as the temporal frequency of the drifting grating increased, and the response peaked at 3.2 Hz, then decreased rapidly as temporal frequency increased further.

For the majority of the units revealing band-pass response (49/58, 84.5%), we calculated both the lower and higher cut-off frequencies at 50% of the maximum response (low_50_ and high_50_ respectively). The median of high_50_ and low_50_ were 6.0 (5.3, 7.0) Hz and 1.40 (1.07, 1.60) Hz (Fig 4B and 4C). The tuning bandwidth was calculated from the difference between high_50_ and low_50_ of each unit, and the median bandwidth of the population 4.70 (4.07, 5.60) Hz (Fig 4D). The histogram in Fig 4E shows the distribution of preferred temporal frequencies for the band-pass population. The preferred temporal frequency of band-pass filtering cells was in the range of 2.0–5.5 Hz, with median 3.2 (2.9, 3.6) Hz. In contrast with previous reports (4.0 Hz in [8]), the population here shows a preference for lower temporal frequencies.

**Fig 5:**
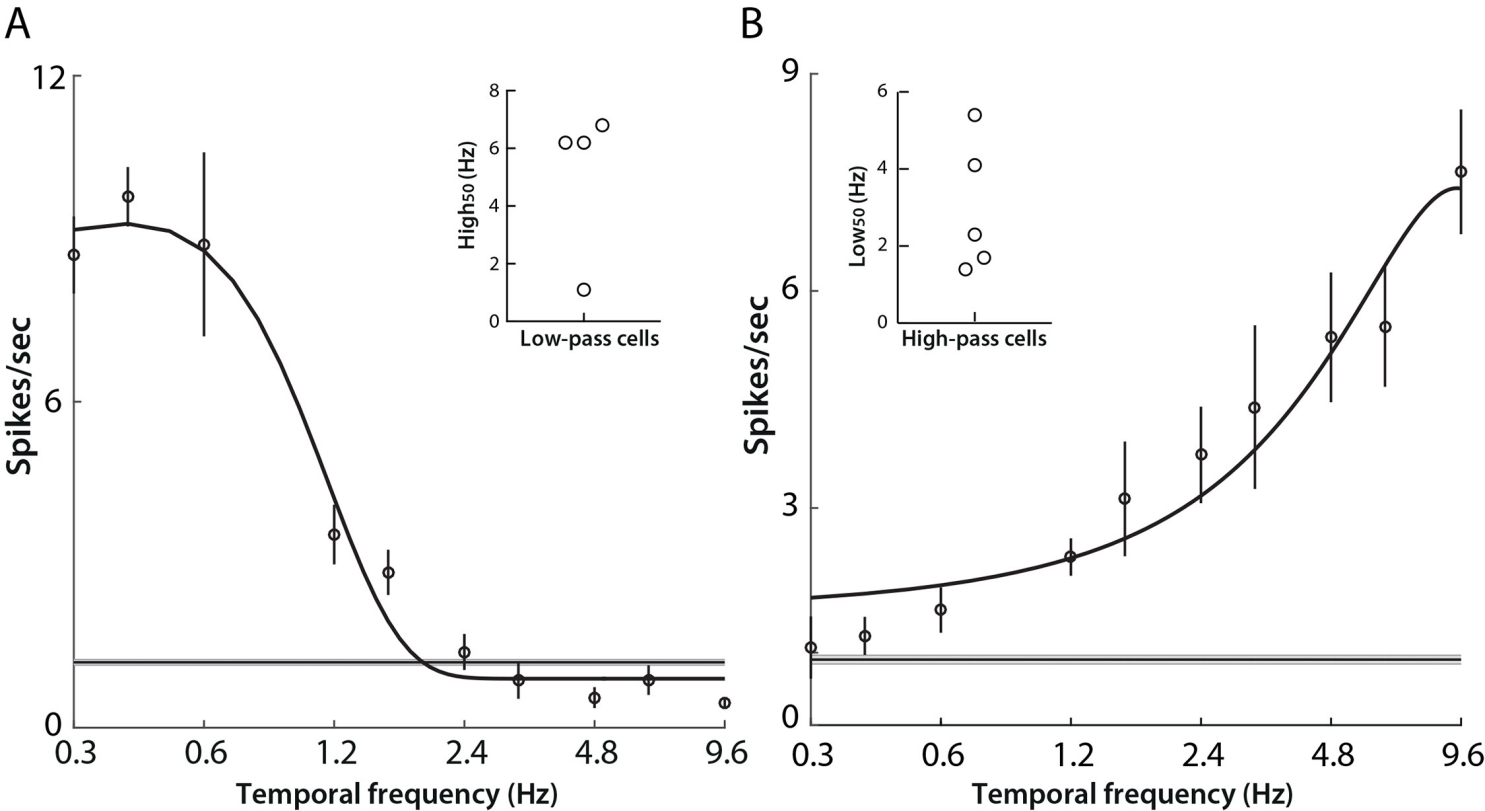
Low-pass and high-pass temporal frequency tuning. A) An example of a cell showing low-pass temporal frequency tuning. Open circles indicate mean firing rates across four repeated presentations of drifting gratings (high_50_ 1.1 Hz). Black curves show the best fits of a two-half-Gaussian function. Grey areas represent SEM of spontaneous activity, with thinner lines indicating mean. Inset: high_50_ of all low-pass cells. B) An example of a cell showing high-pass temporal frequency tuning (low_50_ 1.4 Hz). Inset: low_50_ of all high-pass cells.

Among the 58 units included for temporal frequency tuning analysis, four cells (6.9%) showed low-pass filtering characteristics (Fig 5A), while five cells (8.6%) showed high-pass filtering characteristics with respect to the band of temporal frequencies we presented (Fig 5B). The high_50_ values for the four low-pass units were 1.1 Hz, 6.2 Hz, 6.2 Hz, and 6.8 (inset panel in Fig 5A), whereas the low_50_ values of the five high-pass responsive cells were 4.1 Hz, 2.3 Hz, 5.4 Hz, 1.7 Hz, 1.4 Hz (inset of panel in Fig 5B).

### Linearity

**Fig 6:**
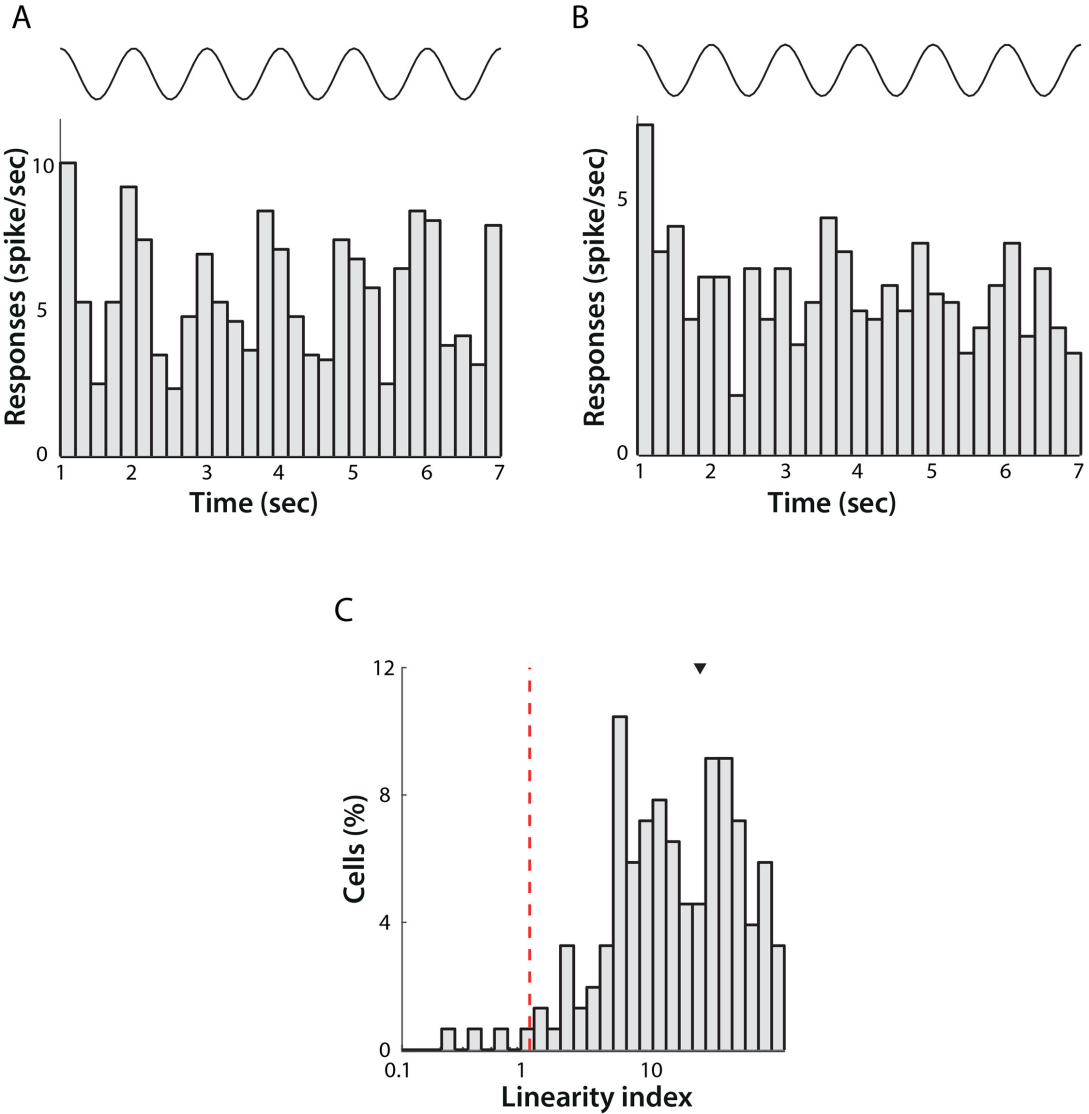
Linearity of LGN neuronal responses. Activity of a single linear (A) and non-linear (B) classified cell across a 7 second presentation of a sinusoidal grating at the preferred spatial frequency of the cell with 1 Hz temporal frequency (average of 6 trials). The top trace represents the time course of the stimulus. The Linearity Index for the linear cell was 1.03 and for the non-linear cell was 0.05. C) Distribution of the Linearity Index across the population of cells. Note logarithmic scale of X-axis. The red dotted line shows the threshold for demarcating linear and non-linear responses. Four cells responded in a non-linear fashion and the remaining 88 were classified as linear in their responses.

Neurons in the mouse LGN have previously been thought of largely as simple relay cells whose responses are amplitude-modulated at the temporal frequency of the stimulus. This response characteristic is also referred to as a linear response. Linearity can be assessed by calculating the ratio between the F1 component at the stimulus temporal frequency and the F0, or DC, component of the response. A value greater than 1 shows a high modulation of the response, or a relatively linear response (Fig 6A), while a value less than 1 corresponds to a response that has little or no modulation at the stimulus temporal frequency, or a non-linear response (Fig 6B).

Overall, the majority of cells displayed linear response characteristics (88/92, 95.6%), as might be expected. Previous studies [8,10] have reported some non-linear cells in their data set, however in even less abundance than we observed.

### Contrast sensitivity

Out of 185 units, 131 were characterized for contrast tuning. Drifting sinusoidal gratings were presented with contrast varying between 0.10 to 0.98 of the maximum screen contrast with a spatial frequency of 0.03 c/deg and 1 Hz temporal frequency. After curve fitting (see Materials and Methods), 128 units were kept for further analysis. For these units, the fitted model explained at least 95% of the data variance.

**Fig 7:**
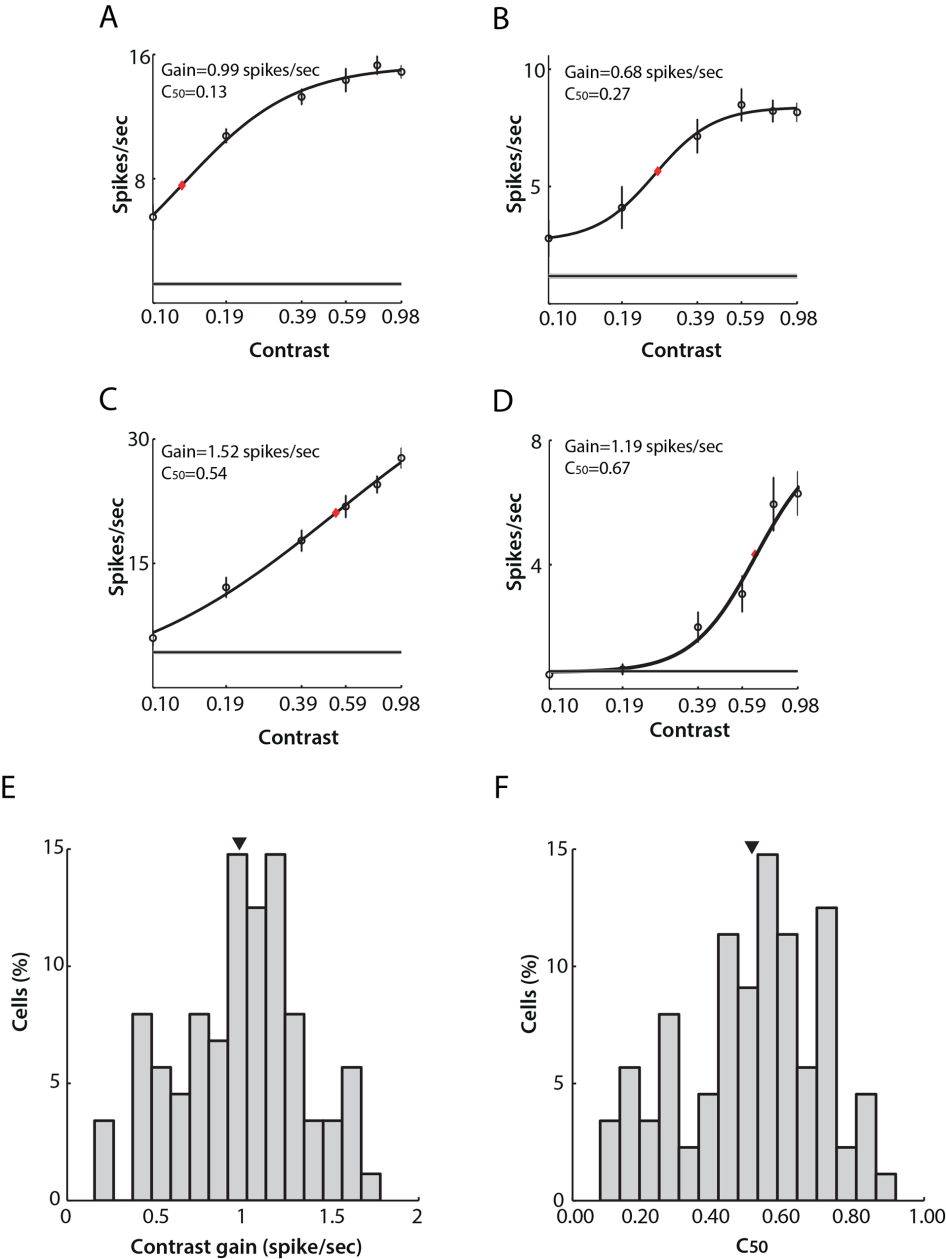
Contrast sensitivity of neurons in mouse LGN. Examples of single cell response tuning to drifting gratings of varying contrasts are shown in A-D. Open circles indicate mean firing rates (average of 10 repetitions). Black curves show the best fits to a hyperbolic function. Red rhomboid indicates C_50_ value. Note logarithmic scale of X-axis. Four types of responses were observed. A) A cell whose response amplitude increased sharply from very low contrast and began to saturate at relatively low contrast. B) A cell showing a sigmoid response curve: response amplitude began to increase rapidly after a short period of slow increase at low contrasts. C) A cell showing an almost linear increase in response amplitude with increasing contrast. D) A cell displaying a linear increase only at higher contrast. E) Distribution of contrast gain across the population. Mean (arrow) ± SEM = 0.98 ± 0.04 spikes/sec. F) Distribution of C_50_ across mouse LGN cells. Mean (arrow) ± SEM = 0.50 ± 0.02.

Generally, the response amplitudes of individual LGN cells increased with increasing stimulus contrast. However, variations from this classical behaviour were found throughout the data set (Fig 7). Some cells (11/128) showed low thresholds of contrast sensitivity, with responses increasing from very low contrast levels and saturating more rapidly (Fig 7A). Some cells (9/128) displayed a sigmoidal response curve (Fig 7B) where response amplitude began to increase rapidly after a short period of slow increase at low contrasts, and then saturated at higher contrast levels. Additionally, some cells (50/128) responded linearly and did not saturate even at the highest contrast (Fig 7C). A further group (58/128) displayed an increased response to gratings of higher contrasts, while not responding to changes in contrast in the lower range (Fig 7D).

Contrast gain and C_50_ were calculated to indicate overall contrast sensitivity across the sampled data. The distribution of contrast gain fell in the range from 0.15 to 1.78 spikes/sec, with mean ± SEM being 0.98 ± 0.04 spikes/sec (Fig 7E). The C_50_ a measure of the contrast level where response amplitude was 50% of its value at full (1.0) contrast, spread widely within the range from 0.10 to 0.98, with mean ± SEM at 0.51 ± 0.02 (Fig 7F).

### Direction/orientation selectivity

**Fig 8:**
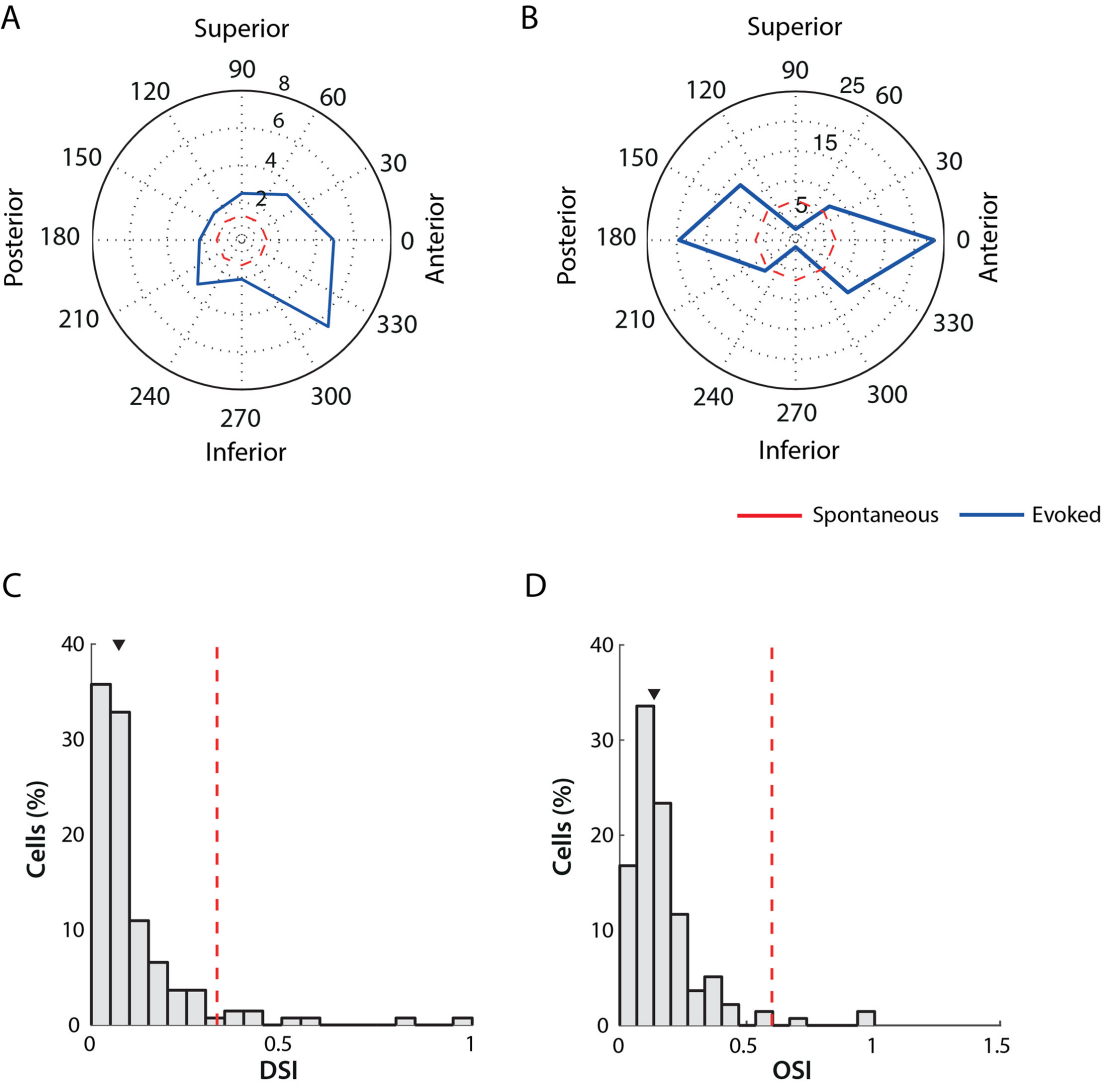
Direction/orientation selectivity in mouse LGN. Panels A and B show examples of single DS/OS neurons that responded preferentially to direction (A), and to anterior-posterior orientation (B). Radial units are spikes/sec. The red dashed line indicates the spontaneous activity level. C) Distribution of Direction-Selectivity Index values. The red dashed lines indicate the threshold for classification (0.33) and the arrow marks the median of the distribution 0.071 (0.038, 0.12). D. Distribution of Orientation-Selectivity Index values. The red dashed lines indicate the threshold for classification (0.6) and the arrow marks the median of the distribution 0.13 (0.082, 0.20).

Piscopo and colleagues [10] recently reported the existence of direction and orientation selectivity in the mouse LGN. We re-examined this property in our data set to see if their findings could be replicated. We measured direction and orientation selectivity from the responses to drifting gratings moving at specific angles between 0 deg and 315 deg. In total, 129 out of 185 cells were characterized with this stimulus class. We report the existence of either direction selective or orientation selective units in the data set. Examples of an individual direction selective unit and an orientation selective unit are shown in Fig 8 panels A and B respectively.

Distributions of DSI and OSI are shown in Fig 8C and 8D. The median DSI was 0.0717 (0.0380, 0.1261) and OSI 0.1332 (0.0824, 0.2060). As previously reported, most cells are not direction or orientation selective. There were, however, 7 cells categorized as DS cells (DSI above 0.33, red dash line in Fig 8C) and 2 cells categorized as OS cells (OSI above 0.6, red dash line in Fig 8D).

### Transient/sustained responses

The temporal profile of each unit’s response was assessed by the Transient/Sustained index (see Methods). Responses to full-field black or white flicker of 600 msec duration were used to quantify the time profile. Cells with index below 1.0 were defined as sustained responsive cells, implying that they responded consistently during the stimulus presentation. Cells with index above 1.0 were defined as transient responsive cells, implying that such cells responded most strongly to the onset of the stimulus and their response decreased thereafter.

**Fig 9:**
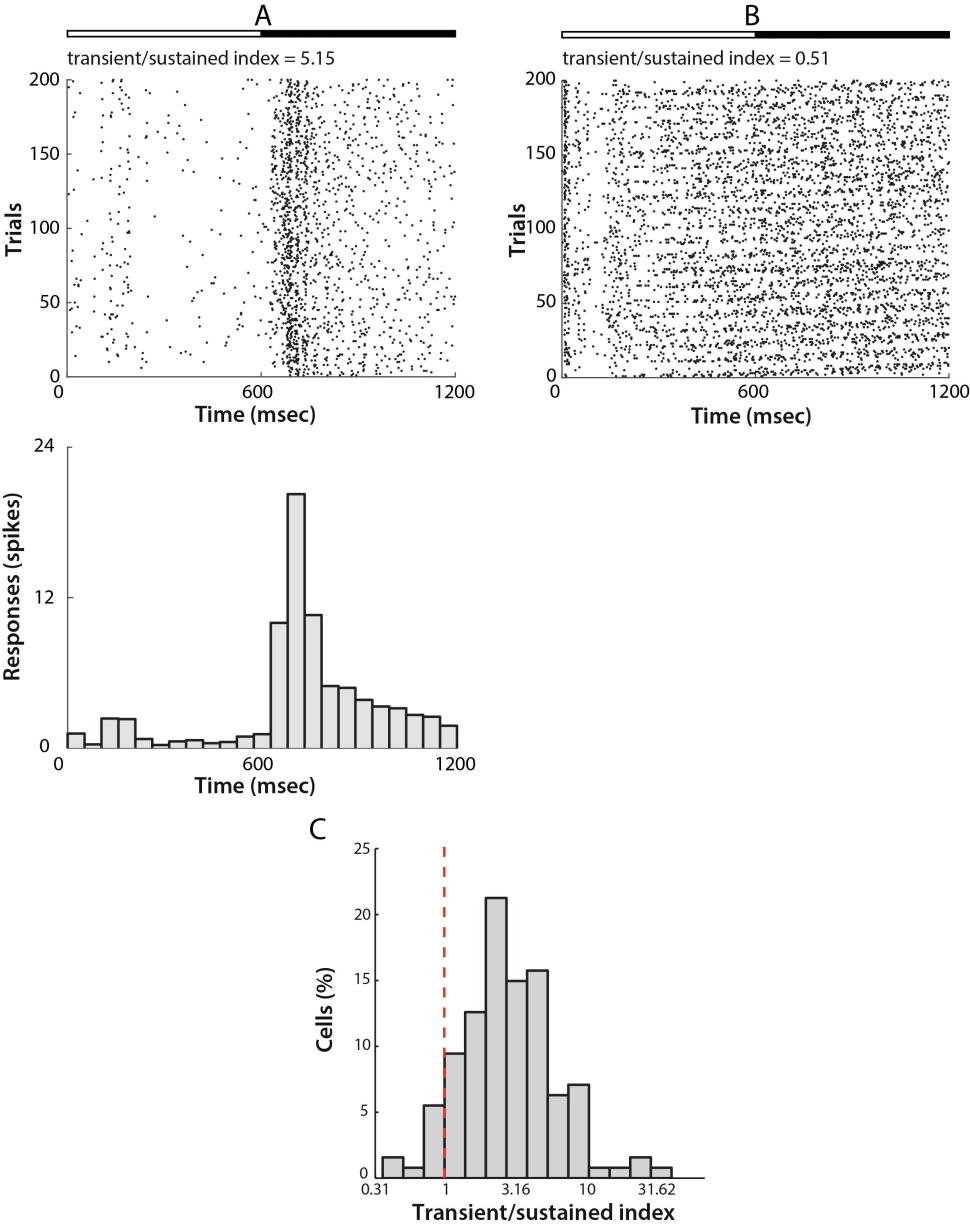
Transient/sustained responses of mouse LGN neurons. A) An example of a cell that responded transiently to a flicker stimulus. The top bar represents stimulus onset, with full-field white stimulation starting at time 0 msec and changing to a black stimulation starting from 600 msec and persisted for another 600 msec. The middle panel shows the raster plot of the cell’s response for all trials represented in the Y-axis against time in the X-axis. The bottom panel shows the PSTH of the same response in 50 msec. bins. B) A cell that responded in a sustained manner to the same stimulus. All parameters as in A. C) Distribution of the transient/sustained index across the population of cells. The red dashed line indicates the threshold for classifying cells as either sustained (<1) and transient (>1). The majority of the cells in our dataset (121 out of 127, 95.3%) responded transiently.

Fig 9A and 9B show examples of single transient and sustained responses to the full-field flicker stimulus, in both cases the top rows show raster plots, while the bottom rows indicate the PSTH across 400 trials. As observed from the PSTHs and rasters, transient cells responded dynamically only at the onset of stimulation, with activity decreasing shortly afterwards (Fig 9A), and some cells even becoming silent. Cells responding in the sustained manner, however, did not exhibit post-excitatory suppression and fired continuously throughout the duration of stimulus presentation (Fig 9B).

Of 127 cells presented with this stimulusthe majority (121/127, 95.3%) were classified as transient (Transient/Sustained Index > 1.0, Fig 9C).

### Parallel projections of ON- and OFF- centre cells

ON- and OFF-centre cells were similarly frequent in our data set, with 48.6% (90/185) ON-centre cells and 51.4% (95/185) OFF-centre cells observed. ON- and OFF-cells overlapped considerably in receptive field sizes, spontaneous and evoked activities.

**Fig 10:**
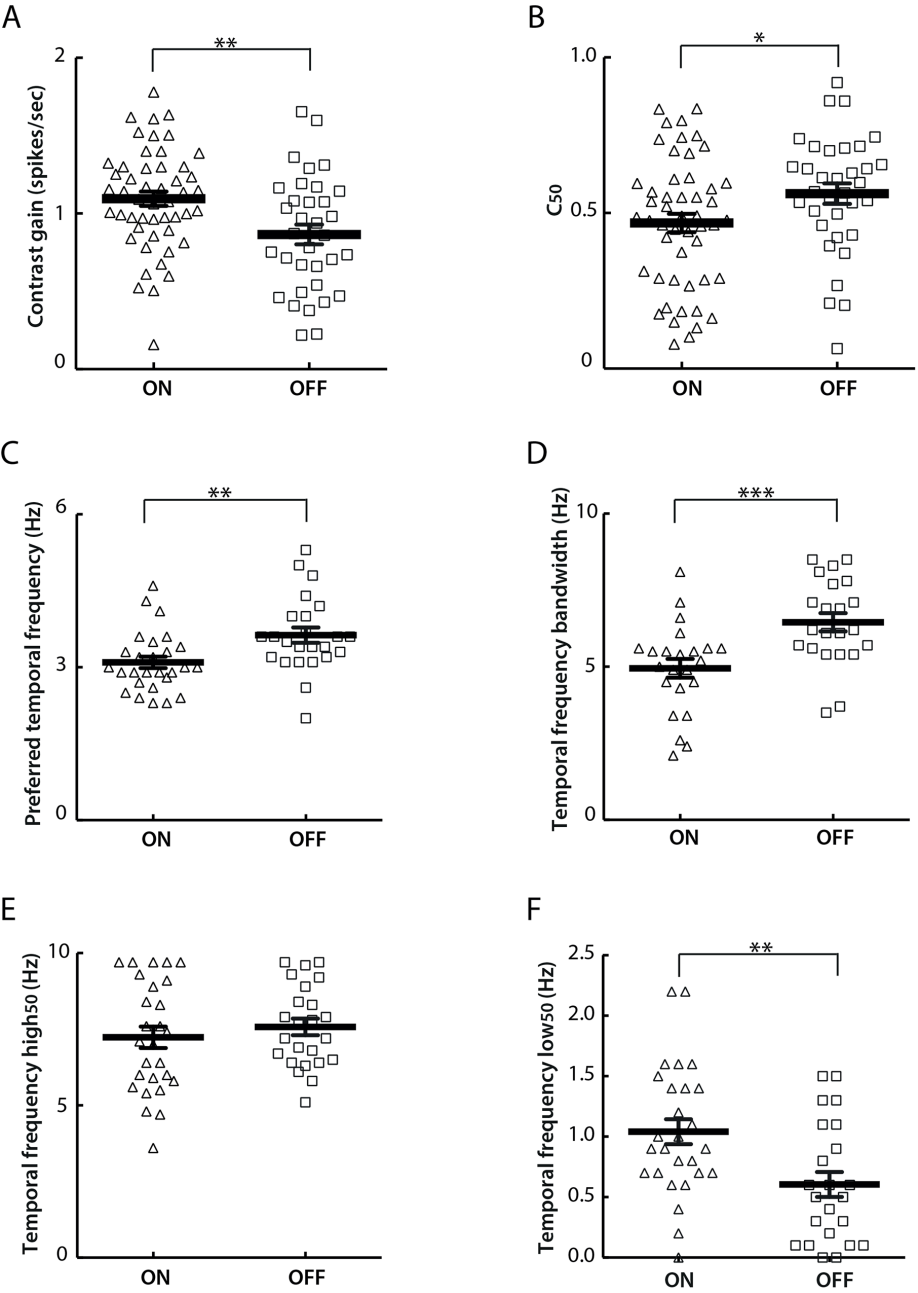
Different response properties of ON- and OFF-centre cells. A) ON-centre cells show significantly higher mean contrast gain than OFF-centre cells (*t*-test, *P*<0.01). ON-centre cells: 1.10 ± 0.05 spikes/sec; OFF-centre cells: 0.87 ± 0.06 spikes/sec. B) ON-centre cells show significantly lower mean C_50_ values than OFF-centre cells (*t*-test, *P*<0.05). ON-centre cells: 0.47 ± 0.03; OFF-centre cells: 0.56 ± 0.03. C) ON-centre cells show significantly lower mean preferred temporal frequencies than OFF-centre cells (*t*-test, *P*<0.01). ON-centre cells: 3.1 ± 0.1 Hz; OFF-centre cells: 3.6 ± 0.2 Hz. D) ON-centre cells have significantly lower mean temporal frequency bandwidths than OFF-centre cells (*t*-test, *P*<0.001). ON-centre cells: 4.9 ± 0.3 Hz; OFF-centre cells: 6.5 ± 0.3 Hz. E) Mean temporal frequency high_50_ values are comparable between ON- and OFF-centre cells (*t*-test, *P*>0.1). ON-centre cells: 7.2 ± 0.4 Hz; OFF-centre cells: 7.6 ± 0.3 Hz. F) ON-centre cells have significantly higher mean temporal frequency low_50_ values than OFF-centre cells (*t*-test, *P*<0.01). ON-centre cells: 1.0 ± 0.1 Hz; OFF-centre cells: 0.6 ± 0.1 Hz. The value for each single ON- or OFF-centre cell is presented as a triangle or a square respectively. The horizontal dark line represents the mean value and the vertical error bar represents ± SEM in each plot.

Interestingly, ON-centre cells were observed to show greater contrast sensitivity than their OFF- centre counterparts in our data set. Specifically, compared to OFF-centre cells, ON-centre cells showed significantly higher contrast gain (ON: 1.1 ± 0.05 spikes/sec; OFF: 0.87 ± 0.06 spikes/sec; *t*-test, *P*<0.01) (Fig 10A), and lower contrast threshold as measured by C_50_ (ON: 0.47 ± 0.03; OFF: 0.56 ± 0.03; *t*-test, *P*<0.05) (Fig 10B).

In addition, ON-centre cells showed a lower degree of temporal frequency selectivity than OFF-centre cells, with significantly lower preferred temporal frequency (ON-centre: 3.1 ± 0.1 Hz; OFF-centre: 3.6 ± 0.2 Hz. *t*-test, *P*<0.01) (Fig 10C), narrower tuning bandwidth (ON-centre: 4.9 ± 0.3 Hz; OFF-centre: 6.5 ± 0.3 Hz. *t*-test, *P*<0.001) (Fig 10D) and higher low_50_ (ON- centre: 1.0 ± 0.1 Hz; OFF-centre: 0.6 ± 0.1 Hz. *t*-test, *P*<0.01) (Fig 10F). The high_50_ of these two subtypes was comparable (ON- centre: 7.2 ± 0.4 Hz; OFF-centre: 7.6 ± 0.3 Hz. *t*-test, *P*>0.1) (Fig 10E).

Apart from the previously mentioned contrast sensitivity and temporal frequency tuning properties, ON- and OFF-centre cells were comparable in other response parameters characterized in this study, including spatial frequency tuning, direction/orientation selectivity, transient/sustained response and response linearity.

### Histological reconstruction of mouse LGN in 3D and cell location mapping

We developed a novel protocol to image Sudan Blue II stained mouse brain in 3D by serial fluorescence tomography performed with an automated microtome (see Materials and Methods). Sudan dyes were applied because they make blocks opaque, leading to blocking of out of focus light and resulting in better resolution in the Z-axis. Staining protocols including Sudan Black, Sudan Purple, and different concentrations of Sudan Blue II were tested. Generally, Sudan Black produced over-strong dye infiltration. Both Sudan Purple and Sudan Blue II were capable of maintaining endogenous fluorescence and limiting out-of-plane fluorescence. Compared with Sudan Purple and higher concentrations (2.5% and 3% in paraffin wax) of Sudan Blue II, the current protocol led to the highest contrast for imaging.

At a thickness of 5 μm per section, we were able to gradually track the small changes in the morphology of the LGN along the anterior-posterior axis, which aided the reconstruction of this nucleus. In total, 290 coronal sections were obtained from one mouse brain spanning the entire LGN. Among them, 280 sections were included for reconstruction. The remaining 10 sections were too vague to outline the LGN boundary both at the beginning of the anterior LGN and towards the end of the posterior LGN.

**Fig 11:**
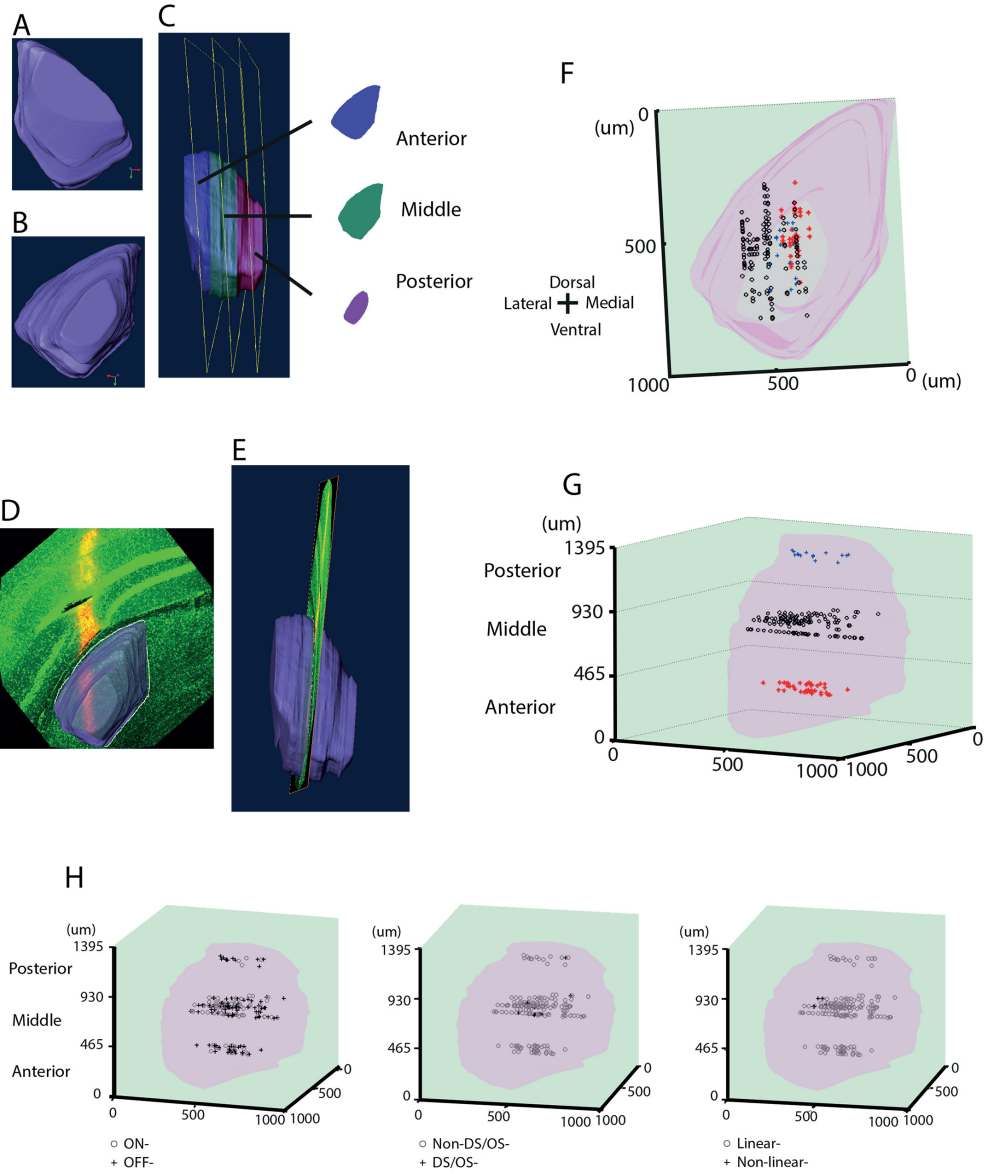
Histological reconstruction of mouse LGN in 3D, with the locations of individual cells. A) The front view of the 3D LGN model. B) The back view of the 3D LGN model. C) The side view of the LGN volume. The model was evenly divided into three sub-regions along the anterior-posterior axis and named as the anterior (blue), the middle (green) and the posterior (magenta) LGN respectively. A representative slice is taken out from each of the subdivision and is shown on the right. D) Mapping of confocal (green) localisation (back view) onto the 3D LGN volume model (outlined in white) along with the electrode tracks in red from one subject to delineate recording sites as belonging to anterior, middle or posterior areas of LGN. E) Side-view of the same mapping. The LGN volume was made transparent to visualize the electrode track. The side view (E) indicates that in this specific recording, the electrode was located in the middle LGN. F) Front view of location of each cell within the LGN volume. G) Side view of panel F. Cell location was determined by mapping confocal image of electrode track and electrode site with the LGN volume. 41, 129 and 15 cells were categorized as located in the anterior (red stars), middle (black dots) and posterior (blue crosses) sub-regions. Scales represent the actual dimensions in μm. H) The three panels show 3D representation within the LGN of ON and OFF cells (first panel), DS/OS cells (second panel), and linear cells (third panel). The exact numbers of these cells are given in Results.

After manual segmentation and further eye-inspection and re-segmentation of unsatisfactory voxels, a finalized LGN volume was obtained that demonstrated a consistent transition in morphology among voxels with smooth borders (Fig 11A–11C). This was taken as the standard 3D histological model of the LGN.

The aim of our development of the LGN volume representation was to classify the locations of electrode tracks within the LGN. To accomplish this aim, the LGN volume was evenly divided into three sub-locations along the anterior-posterior axis, denoted here as the anterior, middle and posterior LGN respectively (displayed as blue, green and magenta in Fig 11C). A representative voxel from each sub-location is shown at right in Fig 11C, which shows distinct morphological features in terms of size, orientation, and boundary, especially medial and ventral borders. These 3D LGN based categorizations together with the targeted coordinates in recording were the criteria for determining the location of electrode.

To localize the electrode track, confocal images were analysed with Amira at the same voxel size as the standard LGN model, with the exception that the thickness on the Z-axis was taken as 1 μm. A continuous comparison between the confocal image and the 3D LGN along the anterior-posterior axis was conducted in terms of morphological features, until the best match was found throughout the LGN volume. A representative example is shown in Fig 11D and 11E. The back view (Fig 11D) displays an almost identical matching of the confocal image with the individual voxel from the LGN model. From the side view (Fig 11E) it is clearly noticeable that the electrode was located in the middle LGN. All of the confocal-imaged electrode tracks from 20 subjects matched well with the 3D LGN model. Moreover, no ambiguous situations, such as electrode track slice falling into adjacent locations (e.g. in the anterior and middle LGN or the middle and posterior LGN) were observed.

By taking advantage of this LGN volume representation to map electrode site, it was also feasible to determine the position of each unit recorded in 3D. This procedure was performed for all 185 units, and the summarized data exported to MATLAB to aid in reconstruction of the recorded units, and the distribution of all units are shown in Fig 11F and 11G. The front view in Fig 11F shows that unit positions spanned the entire LGN along the ventral-dorsal axis, and most units were located in the middle LGN along the medial-lateral axis. Fig 11G displays a side view of the LGN volume (pink), with 41, 129 and 15 units categorized as locating within the anterior, middle and posterior LGN respectively. This categorization of sub-regions resulted from dividing evenly the LGN volume into three along the anterior-posterior axis, and Fig 11G shows that all units could be localised without ambiguity within the different sub-regions of the LGN.

On further investigating the relationship of cell location and response properties we found 14, 72 and 4 ON-cells, and 27, 57 and 11 OFF-cells in the anterior, middle and posterior LGN in that order (Fig 11H—left panel). We only found 8 DS/OS cells in the middle LGN and only one in the posterior LGN (Fig 11H—middle panel). In addition, only one cell showing non-linear characteristics was seen in the anterior LGN while 6 cells from the middle LGN had such property (Fig 11H–right panel). These observations suggest an over-representation of the middle LGN in our dataset that we could attribute to the MEA targeting primarily this region.

## Discussion

### Summary of findings

Electrophysiological results of the morphologically reconstructed LGN cells recorded exhibit a strong centre-surround receptive field organization with field size ranging from 5.3–8.1 deg of visual angle (25th– 75th percentile) and spontaneous activity between 0.9–4.3 spikes/sec (25th- 75th percentile). The majority of such cells also displayed linear summation properties, as measured by the F1/F0 ratio. Our findings on the whole replicate results of previously published reports [8,10]. However, there are a number of important differences. While our temporal frequency tuning results were broadly comparable to previous studies, we did find some cells with “lowpass” (or very low temporal frequency preference) or “highpass” (or very high temporal frequency preference) characteristics. We found direction and orientation selectivity, but in a lower proportion of cells than as described in several recent papers. Our dataset had somewhat more linear response characteristics (higher F1/F0 ratio) than that previously reported. In stark contrast to a previous study [10], we found DS/OS cells to be even more linear (higher F1/F0 ratio) than our general population. We also classified a much higher proportion of cells (95%) as giving transient as opposed to sustained responses to full field flicker. In comparison to an earlier study [8], our LGN cell dataset revealed almost a two-fold increase in average contrast gain response. The distributed nature of cell location in the LGN registered by our novel 3D reconstruction technique with ON/OFF, DS/OS and linear response features is suggestive of the absence of functional topography. Differences between the results of our study and three previous reports are summarised in Table 2, and discussed in more detail below. Taken together with the previous studies, the body of data we have built up on the visual receptive field and stimulus-tuning properties of mouse LGN cells will provide a substantial resource for those seeking to build a computational model of the early visual pathways. This body of data, in combination with the tractability of the murine system for genetic targeting approaches for future experimental work, makes the mouse an attractive system in which to do so, despite its low spatial acuity.

**Table 2:** Comparison of findings from four studies of the LGN.

Finding	Grubb et al [8] ^1^	Marshel et al [9]	Piscopo et al [10] ^2^	Present study ^3^
Recording method	Single electrode	Two photon imaging (OGB-1 AM)	Multi-electrode	Multi-electrode
Anaesthetic	Halothane	Isoflurane	Isoflurane	Isoflurane
RF size (degrees)	5.61 ± 0.41	-	4.9 ± 0.3 ^4^	6.5 (5.3,8.1)
Spontaneous firing rate (spikes/sec)	3.24 (median)	-	1.0 ± 0.28 (sON)	2.0 (0.9,4.3)
			1.7 ± 0.35 (sOFF)	
			1.0 ± 0.35 (tOFF)	
Preferred spatial frequency (c/deg)	0.027 (median)	-	0.05±0.006 (sON)	0.035 (0.031, 0.045)
			0.03±0.003 (sOFF)	
			0.02±0.003 (tOFF)	
Cut-off spatial frequency (c/deg)	0.18 ± 0.01	-	-	0.16 (0.13,0.25)
Preferred temporal frequency (c/sec)	3.95 ± 0.24	-	-	3.2 (2.9, 3.6) ^5^
High cut-off temporal frequency (c/sec)	7.26 ± 0.40	-	-	6.0 (5.3, 7.0) ^5^
Low cut-off temporal frequency (c/sec)	-	-	-	1.40 (1.08, 1.60) ^5^
Linearity (F1/F0)	-	-	1.7±0.06 (sON)	All cells: 13.66 (5.71,34.6)
	(F2/F1 = 0.44 ± 0.04)		1.8±0.12 (sOFF)	DS/OS: 45.6 (9.36, 87.7)
			1.8±0.13 (tOFF)	
			1.0±0.18 (DS/OS)	
Fraction of DS/OS cells	-	23.8%	13%	7.0%
Fraction of Transient cells	50%	-	see Discussion	95.3%

^1^ Mean ± SEM. unless otherwise stated.

^2^ Median ± SE. unless otherwise stated.

^3^ Median (25%, 75% confidence intervals).

^4^ For centre-surround groups denominated sON, sOFF, tOFF.

^5^ For cells classified as bandpass temporal frequency tuned only.

### Methodological Considerations

For stable electrophysiological recording studies, the small volume and deep location of mouse LGN makes precise targeting of this structure difficult, and hence fewer studies have focused on this nucleus than on primary visual cortex. However, recent advances in optogenetic manipulation have created a renewed interest for this structure and to explore its role in visual information processing. Refined MEA targeting procedures combined with a novel 3D LGN reconstruction strategy improved the localization of electrode tracks and recorded cells for this study.

Any visual stimulus response measure can be affected by eye-movement induced retinal-slip and even a small deviation can potentially modulate the outcome [24,25]. A head-fixed anaesthetized animal preparation, as in this study, minimizes such retinal-slip induced cell responses. Wagor et al. [26] though has reported that eye movements are not problematic in quantitative analysis of data from anesthetized mice, even when the ocular muscles are active. Earlier studies in mouse LGN and visual cortex [8,27] have shown that eye movements can shift the location of a receptive field slightly, but that this shift is small in comparison to the average receptive field size. Our receptive field mapping indicated that receptive field locations shifted very little over approximately 2 hrs of recording, consistent with the results reported in an earlier study [8]. Hence, we consider mouse eye movements likely to have had no significant impact on cell responses in our dataset.

### ON/OFF centre-surround receptive fields

ON- or OFF-centre cells were ascertained based on their firing activities to bright stimuli targeting the centre of the receptive field (Fig 1B). Most of the cells recorded were located in the mid-LGN as our MEA—primarily targeted—this region (Fig 1A and Fig 11F and 11G). The median radius of receptive field size in our data was 6.5 deg of visual angle. This value, although proportionally larger than those reported for cat and monkey LGN, is comparable in size to cells from mouse retina and V1 described in earlier studies [14,28] and paralleled the two main studies in mouse LGN [8,10].

In this study we included cells for which we could successfully estimate receptive fields using STA, following Grubb and Thompson [8]. This resulted in keeping approximately 50% of all putative units recorded for further analysis. In contrast, a recent study of mouse LGN, Piscopo et al. [10] used the entire set of putative cells recorded and classified them using a clustering algorithm that lead to the separation of LGN units into 6 subpopulations: sustained-ON, sustained-OFF, and transient-OFF groups (these 3 subgroups included 62.6% of their dataset), direction/orientation selective (10.9%),—suppressed-by-contrast cells (5.4%), and a slow responding group. The last three subgroups lacked robust centre-surround characteristics as revealed by low STA receptive field amplitudes, and instead showed high DS/OS Index, suppressed responses to high contrast, and longer response latencies to slow stimuli respectively. Interestingly, 7.0% (9/129) cells in our dataset exhibited both direction/orientation selectivity and a clear receptive field (Fig 8C and 8D). Our observation does not support the notion that direction/orientation selectivity and ON/OFF receptive fields are entirely separable features in mouse LGN as claimed by Piscopo et al. [10]. We were, however, unable to find any cells that responded in a “suppressed-by-contrast” pattern as reported by Piscopo et al. [10]. This rarely encountered behaviour has been reported from cells in a few other studies: in monkey LGN [29], cat retina [30], and V1 of behaving mice [31]. It might be that this cell type, especially in the mouse LGN, is sparsely distributed, with the majority showing non-classical centre-surround organization and hence, absent from our dataset where all cells analysed had centre-surround receptive fields. Alternatively, this group of cells reported in Piscopo et al. [10] responded “inversely” compared to the rest of the units (i.e., the decrease in baseline activity was of the same magnitude as the increase in firing rate seen in other cells), and it is possible that these cells were interneurons that inhibited responses to incoming stimuli. Nevertheless, it will be crucial to apply quality analysis in single-unit clustering as suggested in Schmitzer-Torbert et al. [32] to uncover the full extent of—different cell types in the mouse LGN.

We also noticed a small number of cells (4/189, 2.1%) from 4 separate recordings that displayed classical receptive field properties, yet could not be driven by any sinusoidal gratings (Fig 2B, cells below the line *y = x*). However, neither their responses to full-field flicker nor their locations within the LGN were different to the rest of the cell population. Similar results were reported in a study on LGN in owl monkeys [33], where 9% of M cells, 6% of P cells and 34% of K cells were not responsive to grating stimuli, yet showed classical receptive field organization. Further analysis revealed heterogeneous responses among these LGN neurons of owl monkeys, including those that only responded to changes in luminance, or moving bars. Response patterns of such LGN cells in mouse to other forms of stimuli may reveal their role in visual information processing.

### Temporal frequency tuning

The majority of LGN cells we recorded showed band-pass temporal filtering characteristics; in a few cases, we observed cells that either had high- or low- pass temporal frequency tuning, or whose tuning preferences fell outside the band of temporal frequencies that we presented (0.3 to 9.6 c/sec). The peak response and high cut-off temporal frequencies of band-pass tuned cells were 3.2 Hz and 6.0 Hz (Fig 4); these values are not inconsistent with the values 4.0 Hz and 7.3 Hz reported by Grubb and Thompson [8]. High- and low-pass filtering characteristics (Fig 5) have not been previously reported in mouse LGN. The small number of low-pass filtering cells (4/57) were obtained from two separate animals, however, their spontaneous firing rates and receptive field characteristics were comparable to those observed in the wider dataset. Similarly, low-pass temporal frequency tuning has been reported in primary visual cortex of monkeys [34] and cats [35]. With the introduction of optogenetic and on-line clustering techniques, one might be able to trace the origins of these cells and target them with specific visual stimulation protocols to elucidate their significance. The diversity of temporal tuning profiles in the dataset may be indicative of distinct temporal-frequency-selective mechanisms as suggested by Foster et al. [34].

### Contrast tuning

Although contrast-tuning curves varied across cells, all cells showed increasing firing rates to increasing contrast. However, the population had a higher contrast gain overall than previously reported, with a mean contrast gain of 0.98 ± 0.04 spikes/sec (Fig 7E) compared to the 0.47 ± 0.05 spikes/sec reported by Grubb and Thompson [8]. This difference may be attributed to the animal’s sex as reported by van Alphen et al. [36]. They argue that contrast sensitivity in mice could vary greatly between sexes and between mice that differ in only a few months of age as responsiveness of the photoreceptors decrease with aging. We only studied female mice aged 2–4 months whereas Grubb and Thompson [8] used mice of both sexes and older than 3 months.

Alternatively, different anaesthetics can lead to distinct contrast sensitivity responses. In primate LGN, Solomon et al. [37] suggested that contrast sensitivity could be altered by different anaesthetics (isoflurane vs. sufentanil). Such observations can also account for differences in contrast gain in mouse LGN, and could potentially explain some of the differences observed with use of halothane [8] versus isoflurane (this study).

### Transient/Sustained responses

One notable difference in our dataset as compared to previous studies was the large preponderance of cells classified as having Transient as opposed to Sustained responses. In our dataset, 95% of cells were classified as having Transient responses. In contrast, Grubb et al. report only 50% of cells as having Transient responses. This may in part relate to methodological differences. We classified cells on the basis of the ratio of their response in the first 50 msec after response onset, to the response during the rest of a 600 msec full screen black/white flicker stimulus. Grubb et al. instead used the correlation between the number of spikes fired and the duration of stimulus presentation, for full screen flashes of varying duration. We applied this metric to a subset of our dataset, but found it to be less reliable than that we adopted. Piscopo et al. did measure a “Sustained Index”, but used it in conjunction with measure of the biphasic nature of spike-triggered average temporal profile, and thus it is not possible to compare with either our results or those of Grubb et al. However, as only 20% of their classified cells fell into the “tOFF” class that they describe, it is likely that their results also reflect a lower Transient/Sustained Index.

### Direction/Orientation selectivity

We observed 9 out of 129 cells (Fig 8A and 8B) that responded selectively to either one direction (DS1>0.33, 7 cells) or two opposite directions of motion (OSI>0.6, 2 cells). OS cells have been reported in marmoset LGN by Cheong et al. [38], and three recent studies have revealed the existence of DS/OS cells in mouse LGN [9–11]. We find similar cells, confirming these earlier findings, and suggesting that mouse LGN cells can encode more diverse signals than was thought until recently. However, it is worth noting one important difference with respect to the DS/OS cells reported by Piscopo et al. [10]: these authors state that the DS/OS cells they found in the LGN were generally nonlinear, whereas the cells that we describe are not only highly linear, but have a larger F1/F0 ratio than our general population. This supports the hypothesis that DS/OS responses in mouse LGN derive from retinogeniculate input, as suggested by Marshel et al. [9].

In our dataset, the location of OS/DS cells observed was primarily in the middle LGN, rather than in the area where DSRGC axons are known to terminate (posterior and dorsolateral shell of LGN) as reported earlier [9–11]. This can be attributed to bias in our MEA insertion towards the mid-LGN region. Other contributing elements for DS/OS response patterns in mouse LGN may include feedback from the superior colliculus and layer 6 in V1, as well as convergence of inputs onto LGN [39–42].

#### Feedback from layer 6 in V1 and the superior colliculus

Retinal inputs comprise 5–10% of synapses onto LGN relay neurons [43,44]. It is therefore plausible that directional/orientation selectivity in LGN derives from non-retinal sources. Zhao et al. [11] and Scholl et al. [45] examined the cortical contribution to this by silencing cortex pharmacologically. They found no changes in the selectivity in LGN neurons, thereby apparently ruling out such a possibility. There is also evidence that superior colliculus neurons exhibit OS receptive fields [46]. However, together with cortical feedback, these sources of input are more likely to be modulators, while retinal inputs provide the primary drive for depolarization in the LGN [47–49].

#### Convergence

In the research conducted by Zhao et al. [11], one third of OS LGN cells were observed to have a single elongated receptive field that could result from the summation of two circular retinal receptive fields with a slight offset in location. This assumption could provide a basis for the selectivity. The other two thirds had circular receptive fields. One possibility could be that LGN cells summed two DSRGCs with opposite preferred direction leading to orientation selectivity [9]. Of the nine DS/OS cells characterized in this study, 33% (3 of 9) of them displayed non-circular receptive fields (receptive field size ratio was out of the range from 0.7 to 1.3). Further connectivity characterization, taking advantage of genetic markers and functional imaging, may be able to clearly distinguish these mechanisms.

In addition, mice display abundant direction/orientation selectivity in V1, especially in layer 2/3 and layer 4 [14,50]. Such prevalence in V1 does not correlate well with the small number of DS/OS cells found in LGN in this study, and raises the question of whether the DS/OS cells in mouse LGN provide inputs to DS/OS cells in V1. Cruz-Martin and colleagues [15] recently addressed this issue with synaptic tracers and noted that DS LGN cells in mice do provide inputs to neurons in the superficial layers in mouse V1. In our dataset 8 out of 9 DS/OS cells responded in a linear manner, supporting their hypothesis considering that the majority of DS cortical neurons in layer 4 are linear as well [14]. However, in contrast, Piscopo et al. [10] found nonlinearity in DS/OS LGN cells.

The existence of DS/OS cells in LGN potentially adds a new dimension to computational capability wherein relay cells in LGN convey information about direction/orientation preference, unlike the standard model in which direction/orientation selectivity is computed anew in the cortex. Although the experiments described here cannot conclusively determine where these DS/OS inputs come from, they provide a valuable starting point for future experimental and computational research.

### Neuronal localization and non-overlapping pathways in mouse LGN

We developed a new technique to reconstruct localization of electrode tracts and cells recorded from within the mouse LGN to very high precision and resolution detailed in the Materials and Methods section (Fig 11A–11C).

Traditional electrolytic lesion protocols along with fluorescent staining and confocal microscopy have been generally used to localise recording regions in the LGN [8,10,11]. Recently, Piscopo and colleagues [10] created a 2D LGN template based on MATLAB scripts to represent each anterior/middle/posterior subdivision and map cell density accordingly.

Numerous studies have attempted 3D visualization of the mouse brain using techniques such as magnetic resonance imaging (MRI) or two-photon laser scanning microscopy. However, such methods lack a satisfactory balance between resolution and volumetric imaging [51,52]. Alternatively, the use of a registration algorithm that aligns 2D consecutive histological slices employing considerable mathematical and computational procedures has to deal with problems of distortion [53,54]. To overcome these issues, we have reported here protocols to perform 3D image visualization of the mouse LGN using a novel standardised LGN model that potentially provides the best outcome for specimen imaging. Moreover, at the resolution of 5 μm/slice, the LGN volume presents a smooth visualization of continuous morphological transition of this structure, and provides a high degree of accuracy in mapping location.

Compared to the 2D simplified templates applied by Piscopo et al. [10], our LGN volume matching model provides a more direct and precise technique in electrode localization (Fig 11D and 11E). Consequently, it provides the feasibility to locate single units in 3D with higher accuracy, linking electrophysiology characterization with anatomical structure (Fig 11F and 11G). Neurons in the dataset showed a higher density in the mid-LGN (Fig 11G) due to the predisposition of MEA targeting this region, while spanning extensively the dorsal-lateral axis (Fig 11F).

We report that ON and OFF electrophysiological responses are distinct in contrast sensitivity tuning and temporal frequency tuning properties (Fig 10), and the differences in contrast response properties are—consistent with the results published by Grubb and Thompson [8]. This finding leads to the question of whether ON- and OFF-cells are differently distributed in anatomy. We therefore addressed this issue by looking into the locations of ON- and OFF-cells separately and noted that these two types were distributed evenly within anterior, middle and posterior areas (Fig 11H left panel). Also, unlike the discovery of regionally biased DS/OS cell in the LGN of rabbits and mice [10,55], the majority of our DS/OS cells (8/9) were located within the mid-LGN (Fig 11H, middle panel). Similarly, we did not observe a clear spatial segregation of the small number of nonlinear LGN cells (Fig 11H right panel). Together, these results suggest that based on the anterior/middle/posterior regional classification, the LGN neurons in mice do not display a well-defined correspondence between anatomical segregation and response properties.
